# Supplementary material for: Order and stochasticity in the folding of individual Drosophila genomes
Source: Nat Commun. 2021 Jan 4;12:41. doi: 10.1038/s41467-020-20292-z (PMC7782554; doi:10.1038/s41467-020-20292-z)
Supplement: Supplementary file 1 — Supplementary Information [file 41467_2020_20292_MOESM1_ESM.pdf]

Supplementary Figure 1

a

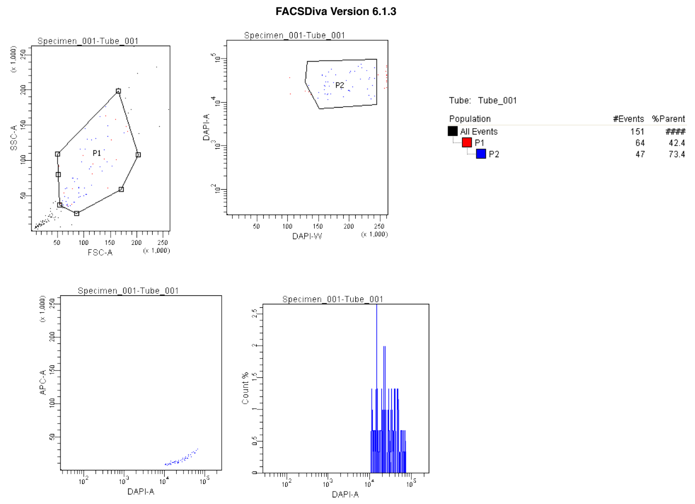

b

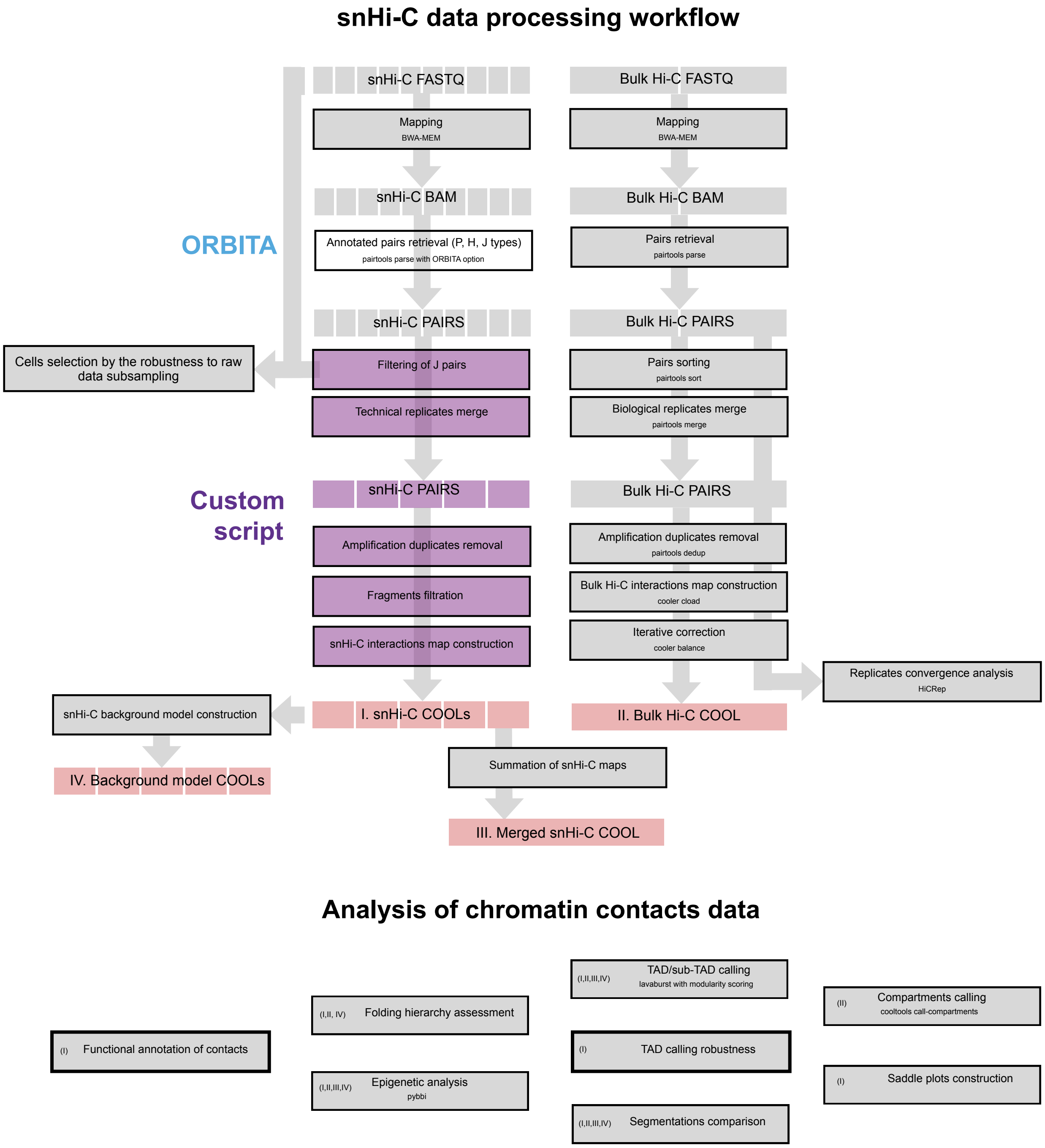

Supplementary Figure 1.
(a) Example of individual nuclei isolation. FACS was performed using DAPI staining. P1 zone contains individual nuclei, and zone P2 contains high-confidence signals. Nuclei were harvested from zone P2. **(b)** Workflow of snHi-C data analysis.

Supplementary Figure 2

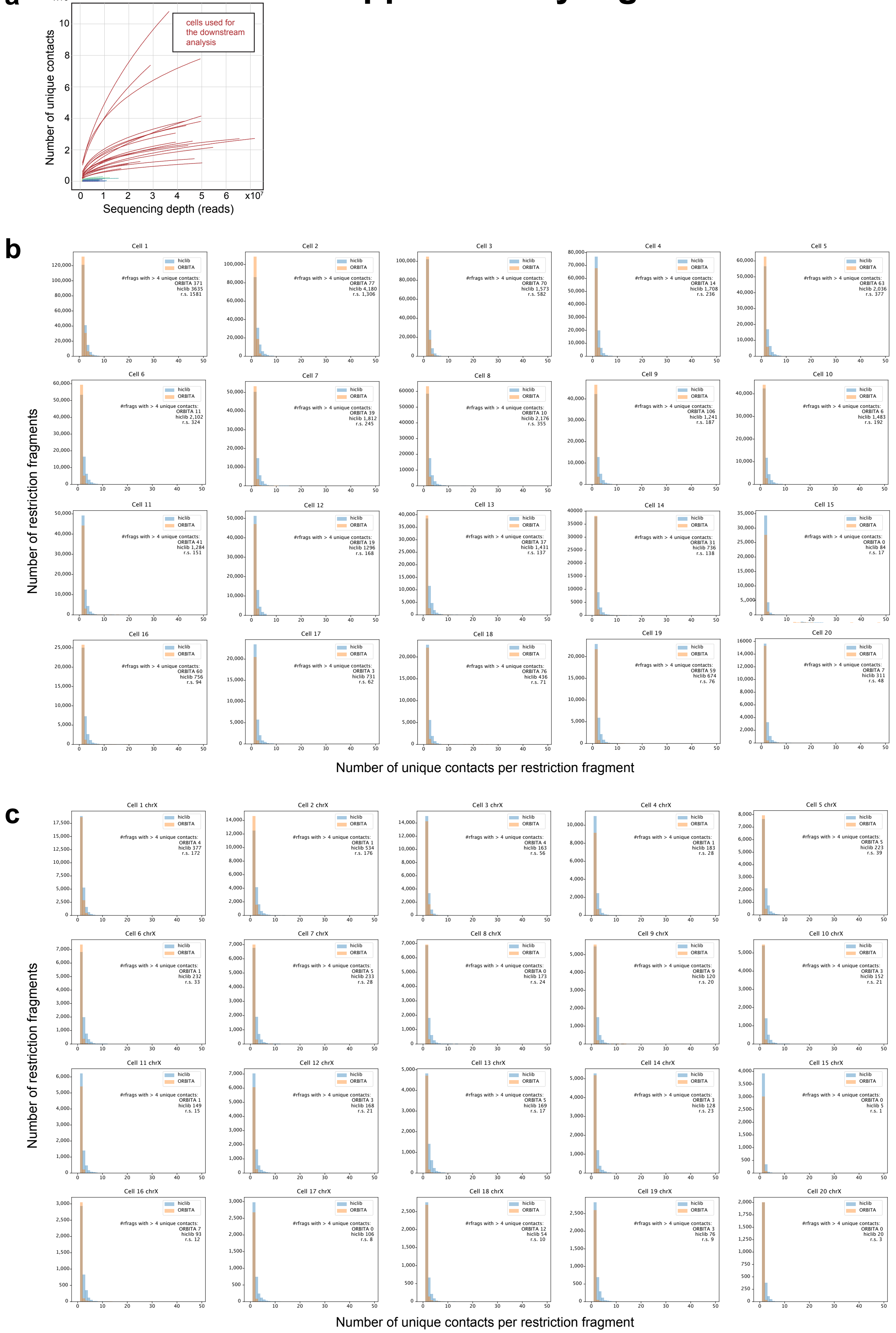

Supplementary Figure 2. Quality control of *Drosophila* snHi-C datasets.
(a) Dependence of number of unique contacts on library size in downsampling analysis (see Methods). The best 20 single cells (red) were selected and then used for additional sequencing. **(b)** Number of unique contacts per restriction fragment (RF) captured by ORBITA (orange) and *hiclib* (blue) for 20 best BG3 snHi-C datasets. Number of restriction fragments with more than four contacts is shown in every plot. Note that the number of contacts called by *hiclib* is larger than the number of contacts called by ORBITA. BG3 is a diploid male cell line; accordingly, in a single nucleus, each RF from autosomes and the X chromosome could establish no more than four and two unique contacts, respectively (see Online Methods for details); r.s., results of downsampling control, averaged over 10 repeat. **(c)** Number of unique contacts per restriction fragment (RF) captured by ORBITA (orange) and *hiclib* (blue) for chromosome X. BG3 cells have only one X chromosome; thus, only 2 unique contacts are possible for a single restriction fragment, corresponding to 2 ends of linear DNA fragment after restriction. r.s., as above.

Supplementary Figure 3

a

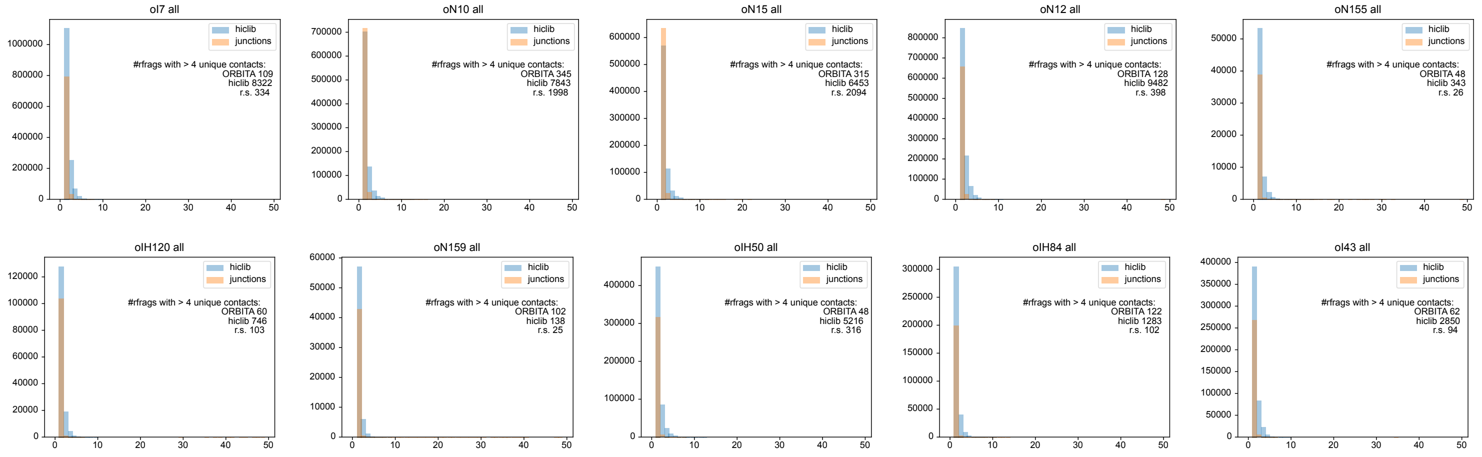

b

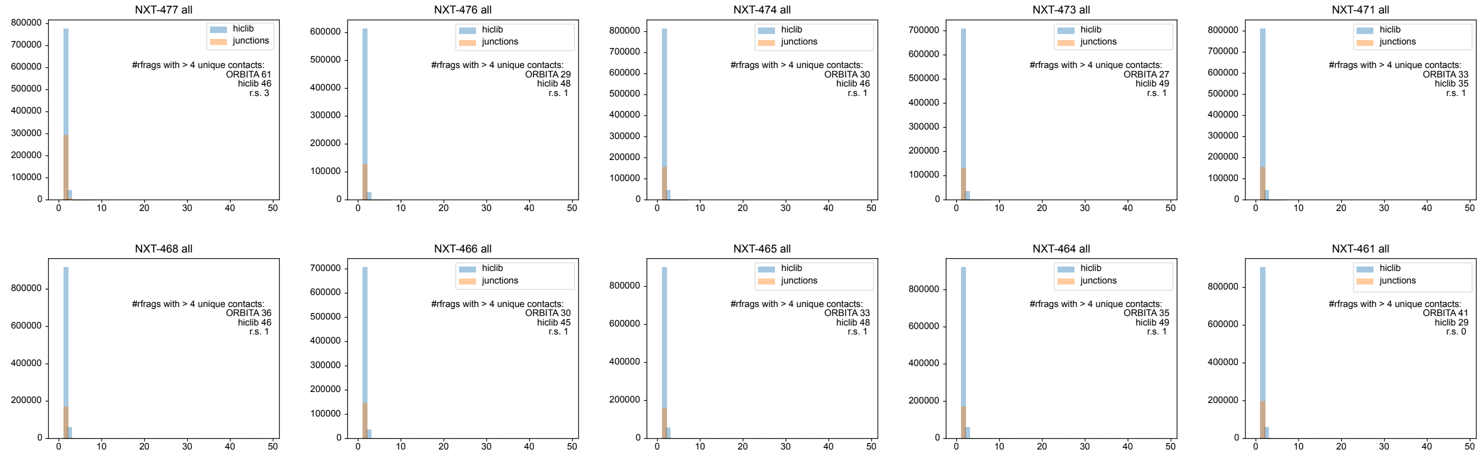

Supplementary Figure 3. Quality control for published mouse snHi-C using ORBITA.
(a) Number of unique contacts per restriction fragment (RF) captured by ORBITA (orange) and *hiclib* (blue) for ten cells from Flyamer et al. (2017)[32]. The cells are named according to the Supplementary Data from Flyamer et al. (2017)[32]. o – oocyte, N – non-surrounded nucleolus, H – Hoechst stain, I – Intermediate, r.s., results of downsampling control, averaged over 10 repeats. (b) Number of unique contacts per RF captured by ORBITA (orange) and *hiclib* (blue) for ten cells from Nagano et al. (2017)[33]. The cells are named according to Supplementary Data from Nagano et al. (2017)[33]; r.s., as above.

Supplementary Figure 4

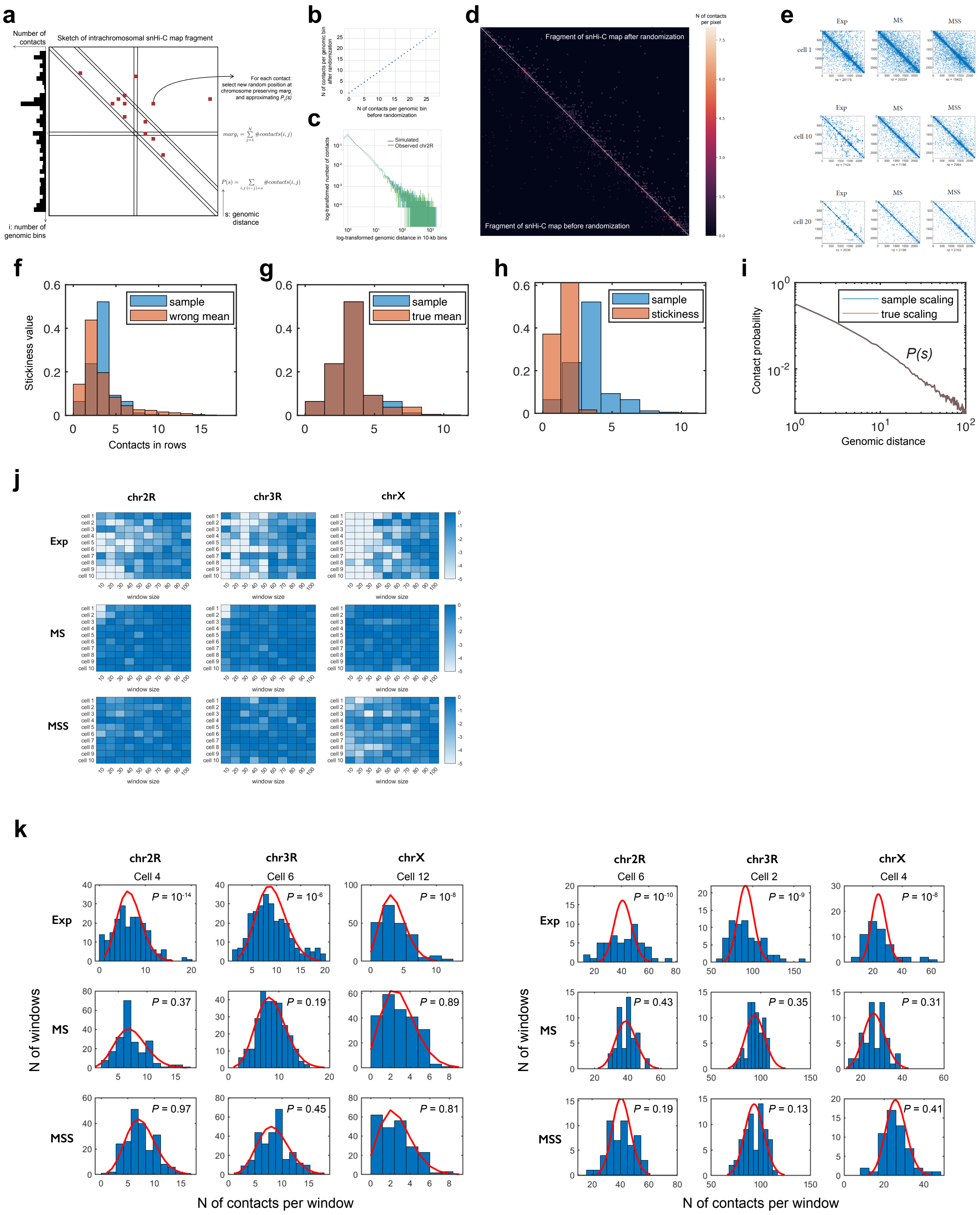

Supplementary Figure 4. snHi-C maps do not follow the rules of random distribution of contacts.
(a) Background model of snHi-C interactions (MSS; Marginal Scaling with Stickiness Model). **(b)** Scatter plot of the initial number of contacts per genomic bin in snHi-C map and after randomization for chr2R of Cell 1 with 107,823 unique contacts. **(c)** Contact probability $P_c(s)$ for chr2R of Cell 1 before (green) and after (blue) randomization. **(d)** Cell 1 snHi-C interactions map for a region of chr2R (lower triangle) and randomized background control (upper triangle) (see Methods). Note the presence of contact clusters at the diagonal both in original and reshuffled data. **(e)** Examples of experimental (Exp) single-cell Hi-C maps with those simulated using the MSS and MS models. **(f-h)** Derivation of the stickiness values (Y axis) given the coverage of bins (numbers of contacts in rows, X axis) obtained by iterative approximations for the MSS model and chr2L (merged snHi-C data were used). n = 2,302 bins. **(f)** Histograms of observed coverage from merged snHi-C map (blue) and of theoretical values (brown) calculated with (red) at the first step of the iterative procedure; wrong mean – computed with wrong stickiness. **(g)** The same histogram as in (f) after a series of iterative corrections of the stickiness values that led to convergence towards the limiting values. The resulting distribution of the coverage (red) reproduces the experimental values; true mean – computed with true stickiness, which is the outcome of the iterative procedure. **(h)** Distributions of the experimental coverage (blue) and of the limiting stickiness (red) are significantly different. Notably, the stickiness values have lower variance than the experimental coverage because the latter incorporate fluctuations of the contact probability. **(i)** Initial and limiting scaling probability functions (see Methods) remain unchanged after the iterative approach. **(j)** Heatmaps of log10 of *p*-values for the test for the top-10 cells sorted to their contact densities. Clustering of contacts at the scale of TADs cannot be explained by the random models at the significance level. **(k)** Experimental, MS, and MSS distributions of the number of contacts in windows of the size bins (100 kb) (left) and bins (400 kb) (right) displaced at the main diagonal and their best Poisson distribution (in red). *P*-values are calculated in Chi-Square Goodness of Fit Test. Left: n = 211, 279 and 224 windows for 2R, 3R and X, respectively. Right: n = 52, 69 and 56 windows for 2R, 3R and X, respectively.

Supplementary Figure 5

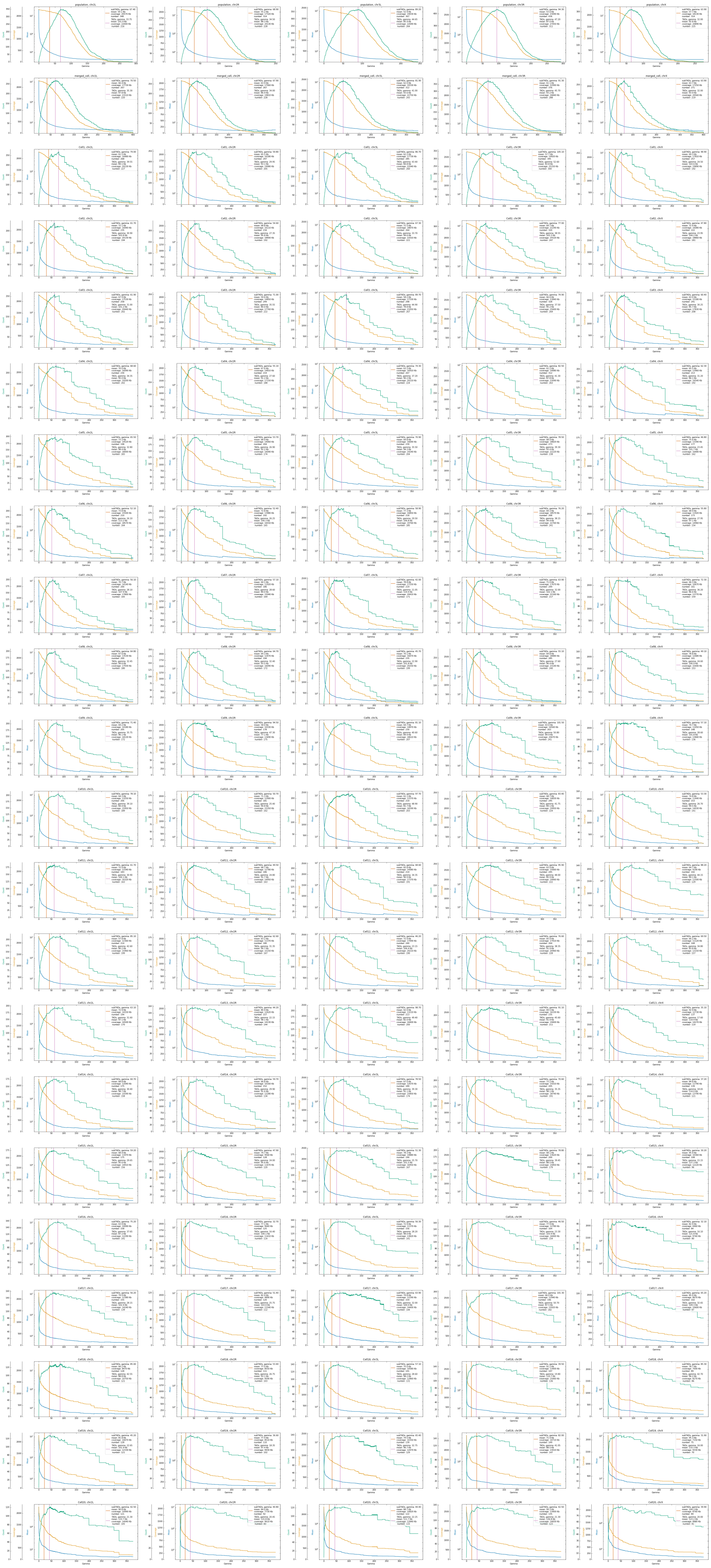

Supplementary Figure 5. Selection of TAD calling parameters.
Dependence of mean TAD size, genome coverage with TADs, and number of called TADs on the γ parameter value for all analyzed chromosomes for bulk in situ Hi-C data, merged dataset, and all single cells. Iterative correction of the maps was used prior to TAD calling for the bulk *in situ* Hi-C dataset. Count – number of TADs identified; Coverage – coverage of the genome with the TADs identified; mean – mean TAD size. In the inset: number – number of TADs and sub-TADs identified.

Supplementary Figure 6

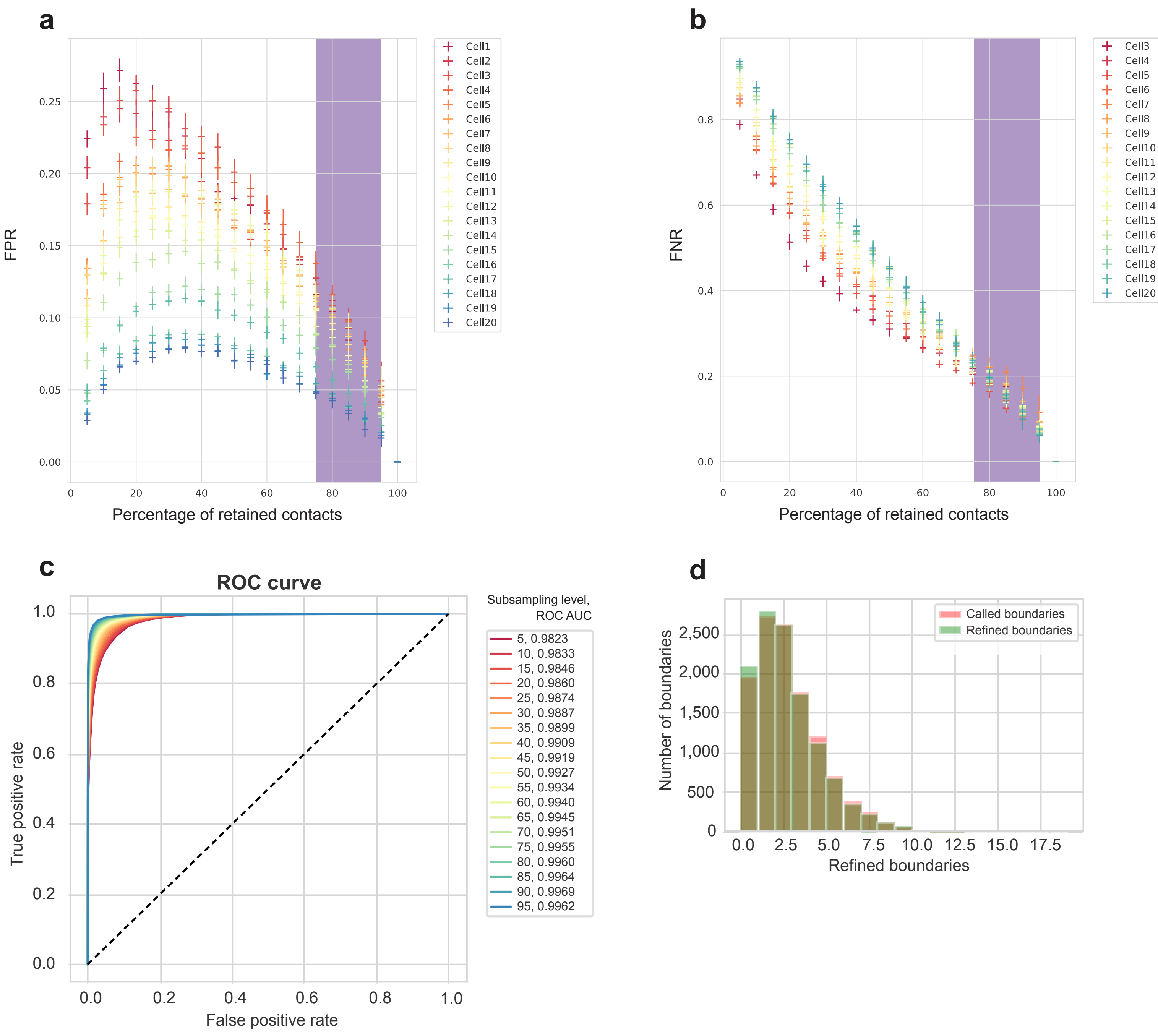

Supplementary Figure 6. TAD calling robustness to sampling procedure.
(a-b) Mean ratio of overrepresented boundaries (false positives) out of true non-boundary genomic bins (false positive rate (FPR)) (a) or underrepresented boundaries (False Negatives) out of true boundaries (false negative rate (FNR)) (b) for TAD calling on subsampled snHi-C maps (see Methods). The snHi-C maps were independently subsampled by 5% levels from the initial contacts of each dataset. Ten subsampling iterations per dataset and subsampling level were performed. Violet rectangle shows the diapason of data downsampling used for testing of TAD boundary robustness to sampling procedure.
(c) True TAD boundaries were predicted based on the collective support by different subsampling levels and iterations (see Methods). ROC curves for different threshold subsampling levels are shown. Subsampling level 95 corresponds to 95% of initial contacts per dataset; level 90 corresponds to collective support from 95% and 90% subsampling iterations etc. Level 90 was selected as the threshold. The following final criteria were selected: collective support is smaller than 45% at 90–95% subsampling levels. The resulting accuracy is 0.9765.
(d) Distribution of number of cells in which the boundary is present. Out of 9,942 initially called boundaries across all the cells and chromosomes, 9,788 are confirmed by subsampling analysis.

Supplementary Figure 7

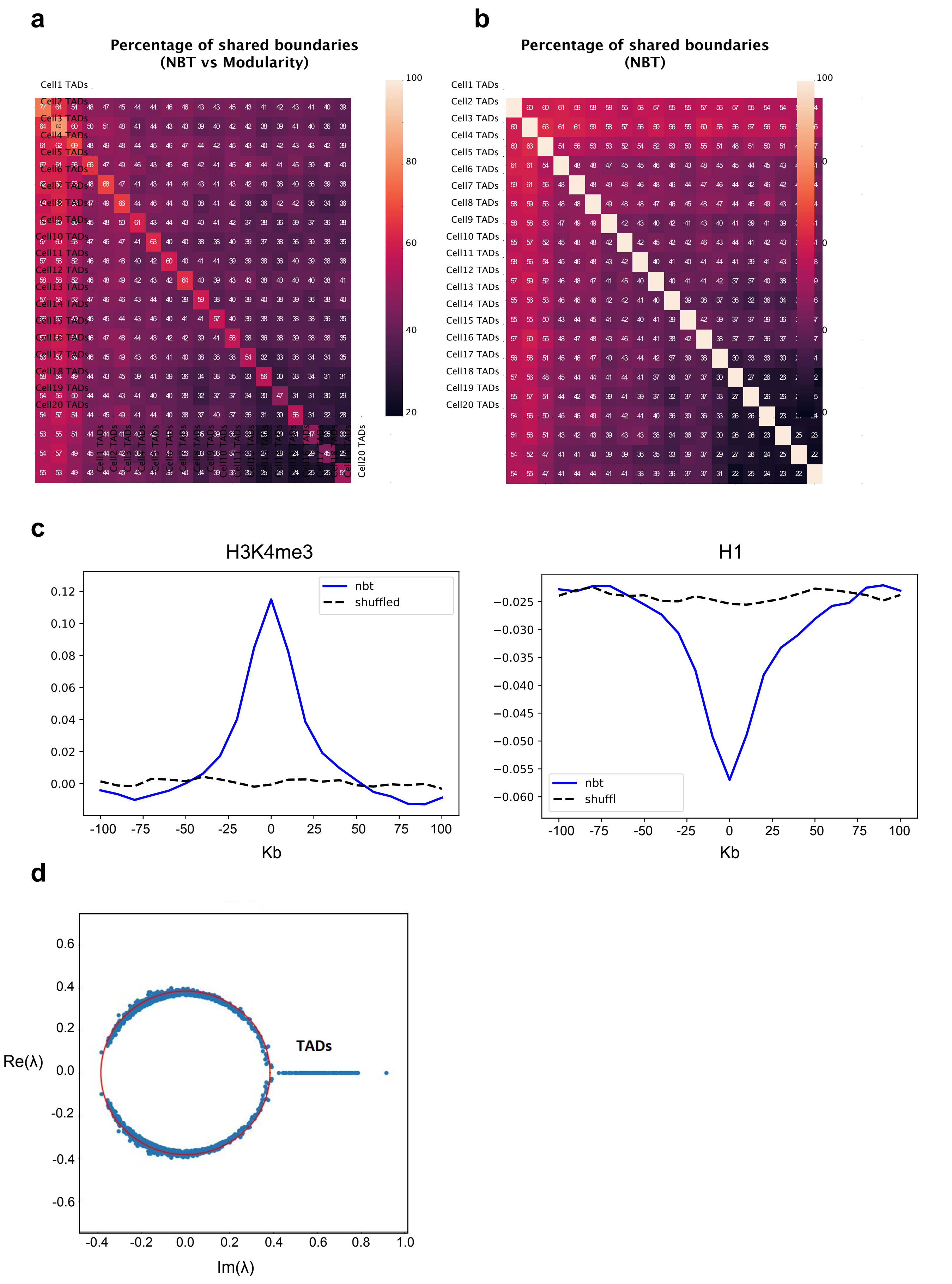

Supplementary Figure 7. NBT as an alternative approach for identification of TAD boundaries.
(a) Percentage of TAD boundaries shared between NBT- and modularity-derived TAD segmentations in individual cells. The mean percentage of shared boundaries is 61%. **(b)** Percentage of TAD boundaries shared between single cells for the NBT TAD calling procedure. The mean percentage of shared boundaries is 42%. **(c)** Epigenetic profiles around the NBT-identified TAD boundaries. **(d)** Spectrum of the non-backtracking operator for cell 3, chr3L. The corresponding constraining disk of the radius r_c for the stochastic block model is shown by red.

Supplementary Figure 8

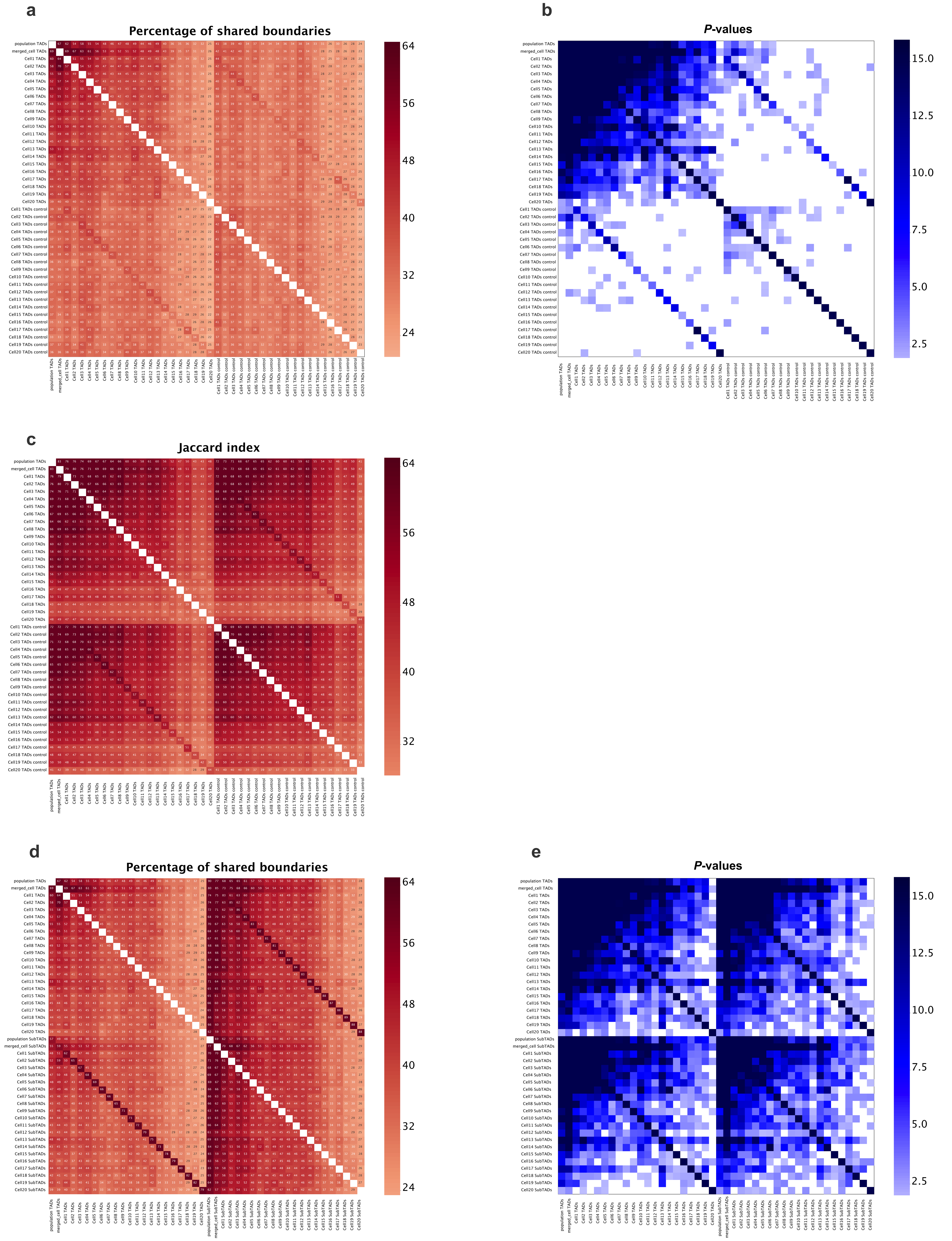

Supplementary Figure 8. TAD boundaries are shared between individual *Drosophila* cells.
(a) Percentage of TAD boundaries shared between *Drosophila* BG3 single cells, bulk BG3 *in situ* Hi-C, and merged snHi-C data, pairwise comparisons. The mean percentage of shared boundaries between individual cells is 39.45%. On average, 46.6% of population-identified TAD boundaries are present in the single cells. In control maps with shuffled contacts (lower part of the plot) preserving marginal distributions and scaling, only 34.95% of boundaries are shared with population boundaries, and 32.47% of boundaries are shared between pairs of shuffled maps (see Methods). **(b)** P-values of permutation tests for the TAD boundaries from (a). Permutation tests were performed 1,000 times (see Methods). -log10 values are shown. **(c)** Jaccard index of shared TAD regions between *Drosophila* BG3 single cells, bulk BG3 *in situ* Hi-C, and merged snHi-C data, pairwise comparisons (see Methods). **(d)** Percentage of TAD boundaries shared between *Drosophila* BG3 single cells, bulk BG3 *in situ* Hi-C, and merged snHi-C data, TAD and sub-TAD, pairwise comparisons. **(e)** *P*-values of permutation tests for the TAD and sub-TAD boundaries from (d). Log10 values are shown.

Supplementary Figure 9

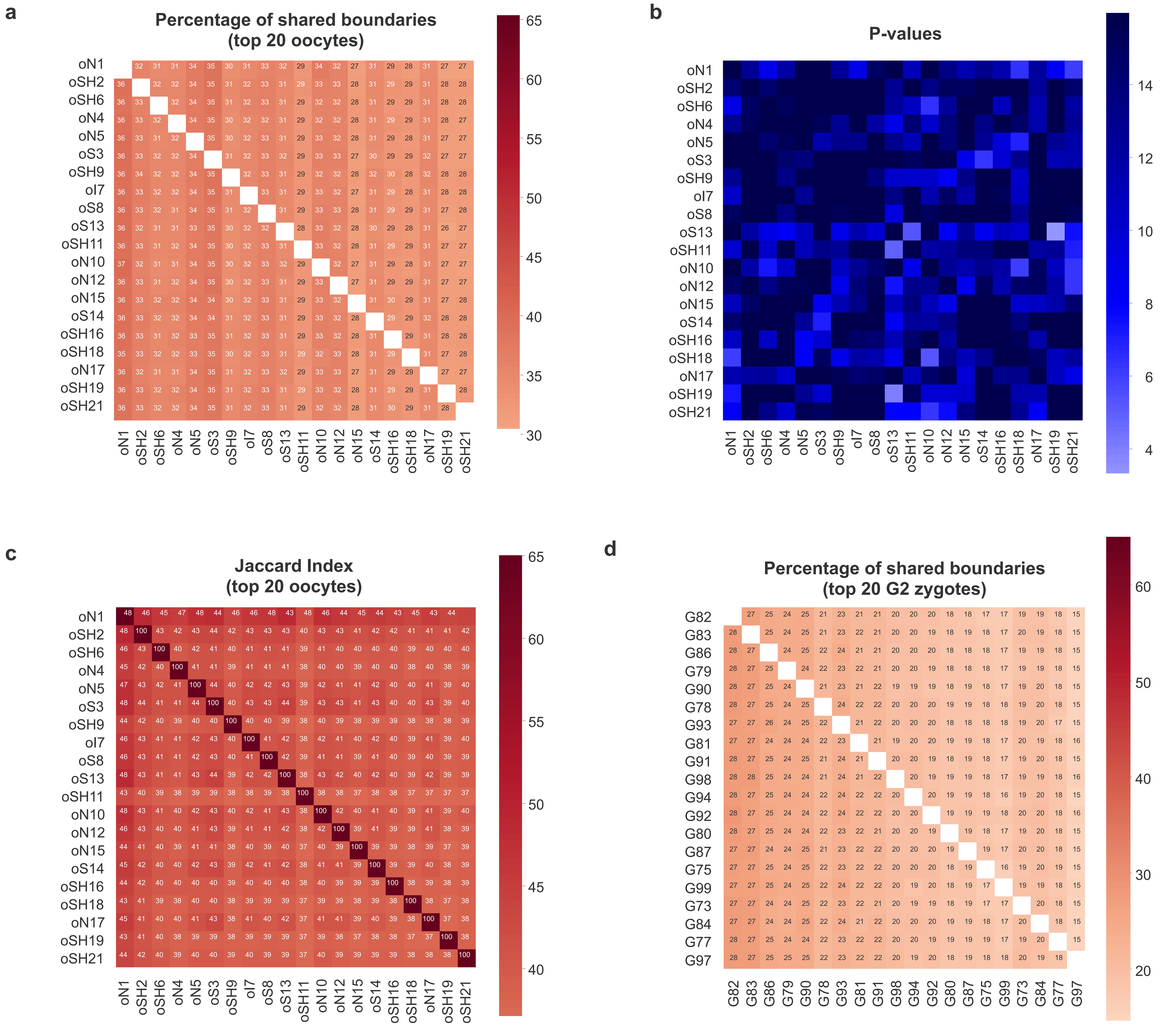

Supplementary Figure 9. TAD boundaries are less stable between mouse cells.
(a) Percentage of TAD boundaries shared between murine oocytes single cells[32] (top-20 cells based on the number of contacts), pairwise comparisons. The mean percentage of shared boundaries is 31.2%. **(b)** *P*-values of permutation tests for the TAD boundaries from (a). Permutation tests were performed 1,000 times. -log10 values are shown. **(c)** Jaccard index of shared TAD regions between mouse single oocytes[34], pairwise comparisons. TADs were called with the procedure similar to the TAD calling in *Drosophila*, as described in Methods, varying gamma value from 0 to 375 during step 1. **(d)** Percentage of TAD boundaries shared between murine G2 zygotes pronuclei (top-20 pronuclei based on the number of contacts), pairwise comparisons. The mean percentage of shared boundaries is 21%.

Supplementary Figure 10

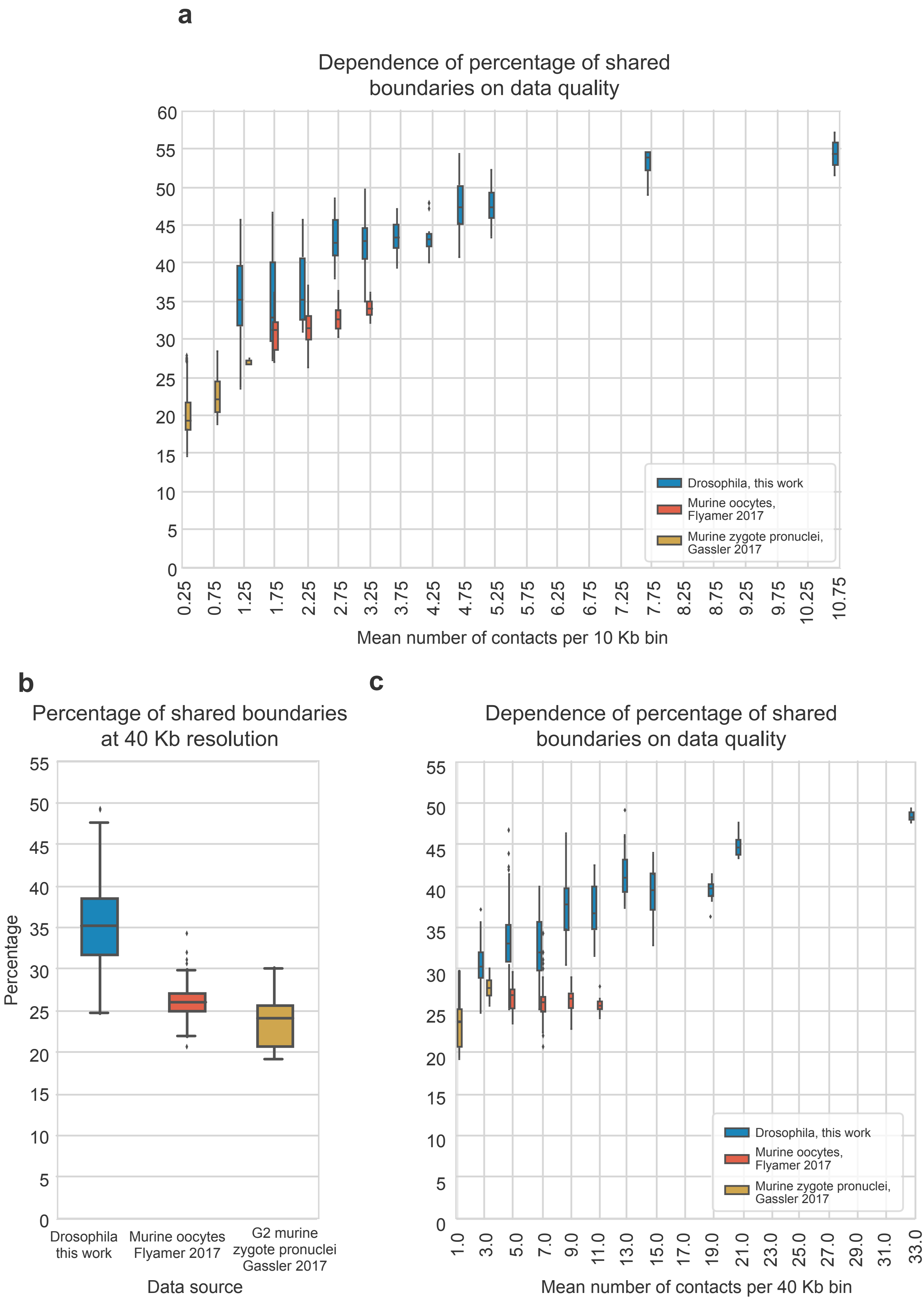

Supplementary Figure 10. Different stability of TAD boundaries between mouse and *Drosophila* is robust to the data resolution selection and data quality.
(a) Percentage of shared boundaries for different levels of data quality, as assessed by mean number of contacts per 10-kb genomic bin per dataset. Boxplots represent the median, interquartile range, maximum and minimum. Total n = 380 cell-to-cell pairwise comparisons for each of three types of boxplots in the analysis. Related to Fig. 3e. **(b)** Percentage of shared boundaries between all pairs of cells of *Drosophila* in this work, top-20 oocytes from Flyamer et al. (2017)[32] and top-20 G2 zygote pronuclei[34] at 40 kb. Boxplots represent the median, interquartile range, maximum and minimum. Total n = 380 cell-to-cell pairwise comparisons for each of three types of boxplots in the analysis. **(c)** Percentage of shared boundaries for different levels of data quality, as assessed by mean number of contacts per 40-kb genomic bin per dataset. Boxplots represent the median, interquartile range, maximum and minimum. Total n = 380 cell-to-cell pairwise comparisons for each of three types of boxplots in the analysis. Related to Supplementary Fig. 10b.

Supplementary Figure 11

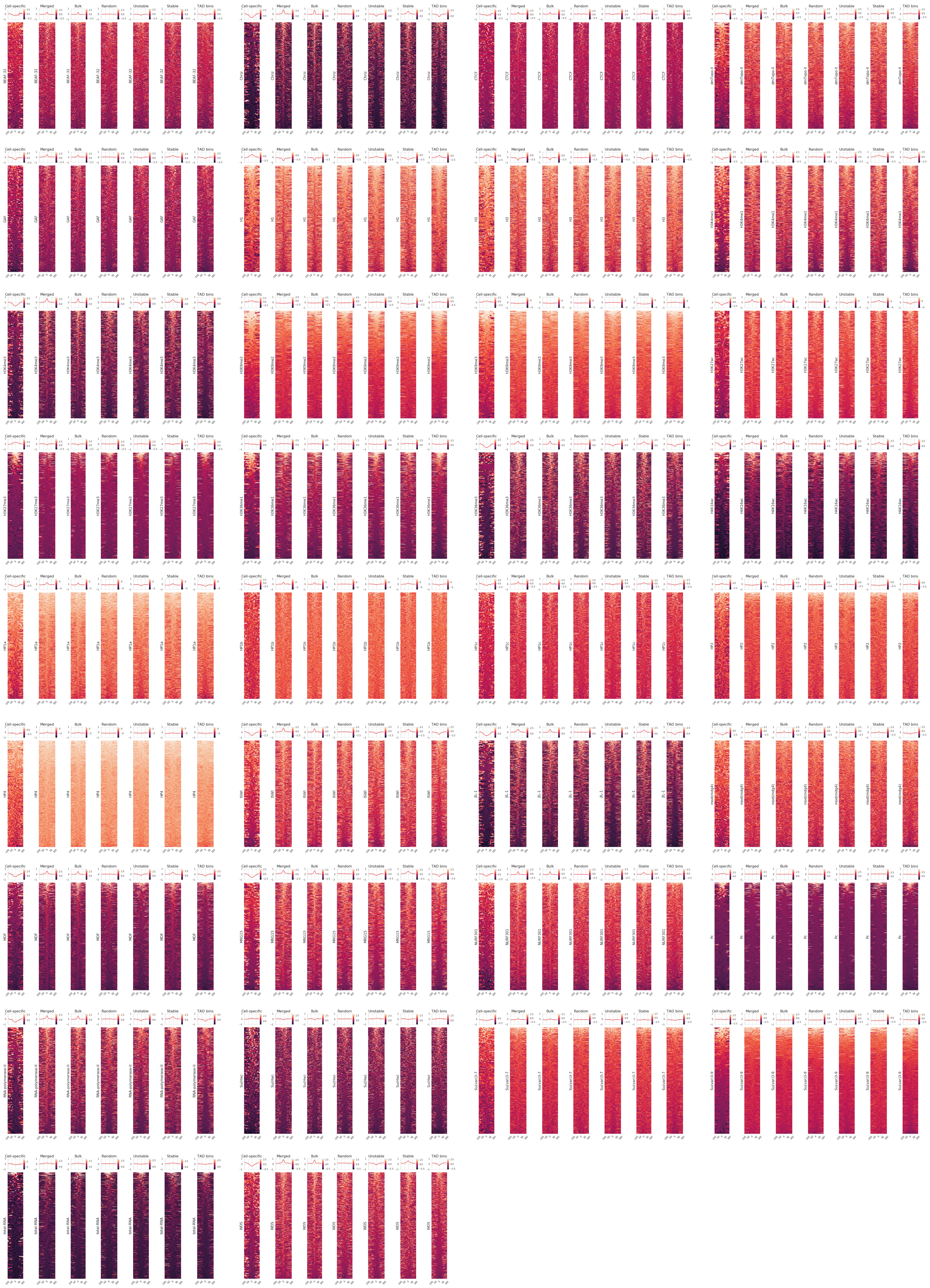

Supplementary Figure 11. Epigenetic properties of different types of TAD boundaries.
Heatmaps with z-score (upper panel) of selected chromatin marks centered at single-cell TAD boundaries from different groups (+/- 100 kb). Bulk – conventional BG3 *in situ* Hi-C; merged – aggregated snHi-C data from all individual cells; stable – boundaries found in more than 50% of cells; unstable – boundaries found in less than 50% of cells; cell-specific – boundaries identified in any one individual cell; TAD bins – genomic bins from TAD interior; random – randomly selected genomic bins. TAD boundaries are refined by the subsampling robustness protocol (see Methods).

Supplementary Figure 12

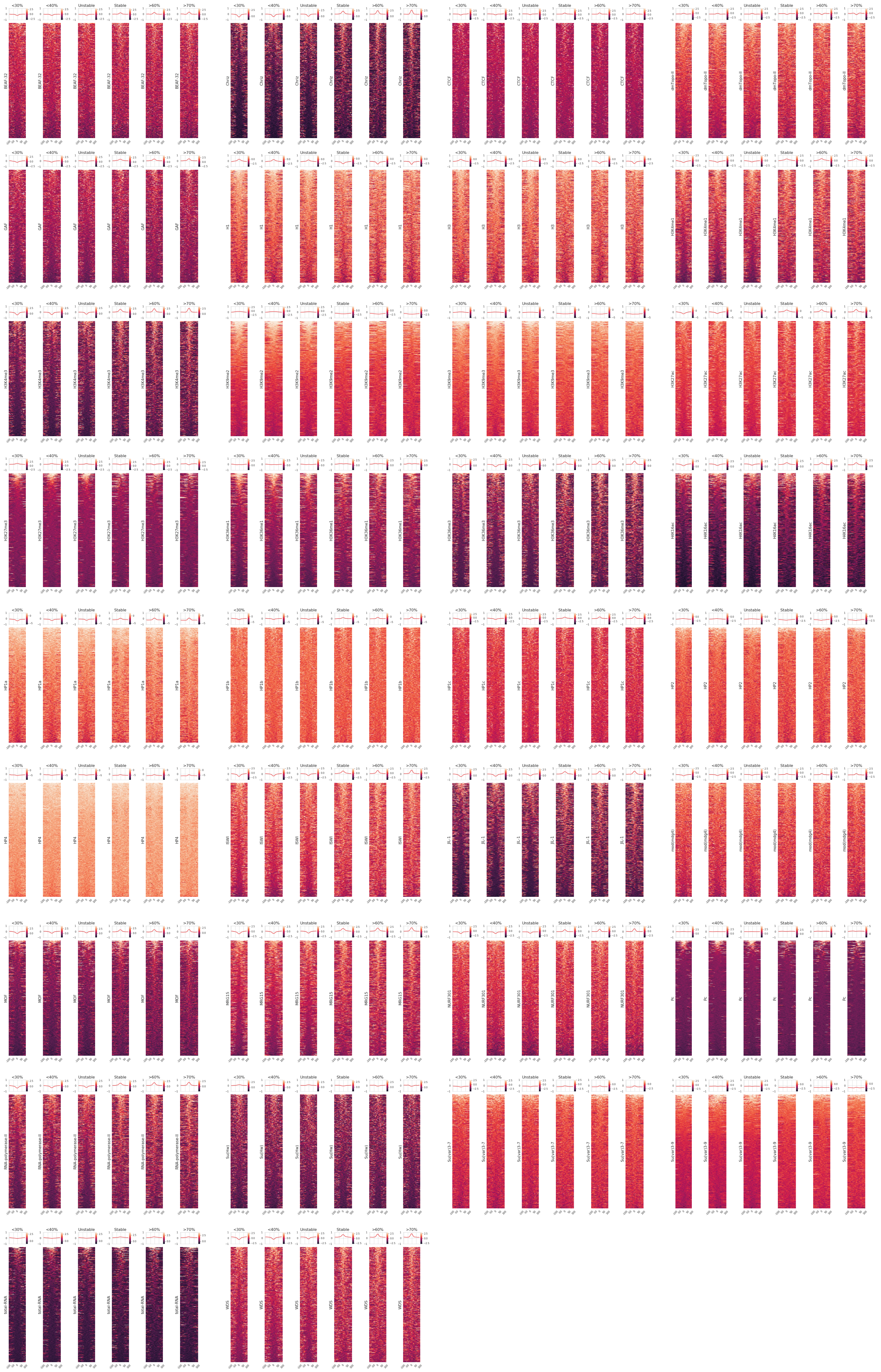

Supplementary Figure 12. Epigenetic properties of different types of TAD boundaries (related to Supplementary Figure 11; stable – boundaries found in more than 60% of cells; unstable – boundaries found in less than 40% of cells).

Supplementary Figure 13

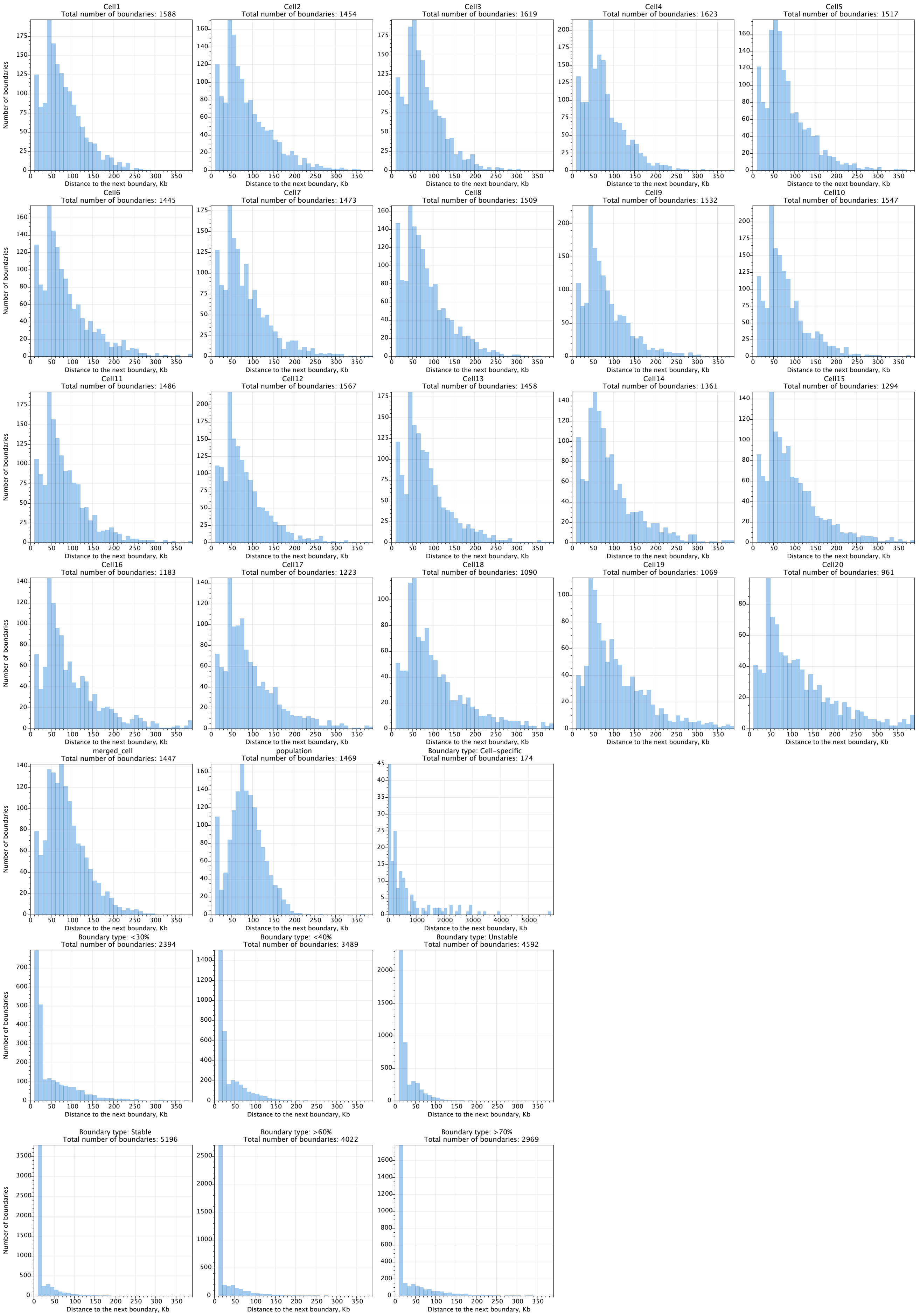

Supplementary Figure 13. Distributions of distances between boundaries of different types and the number of boundaries. Rows 1-4 represent the boundaries from individual cells. Rows 5-7 represent the boundaries from the bulk Hi-C data, merged datasets, alongside cell-specific boundaries, unstable and stable boundaries. In all the cases except cell-specific boundaries, the distributions are demonstrated up to 400 kb.

Supplementary Figure 14

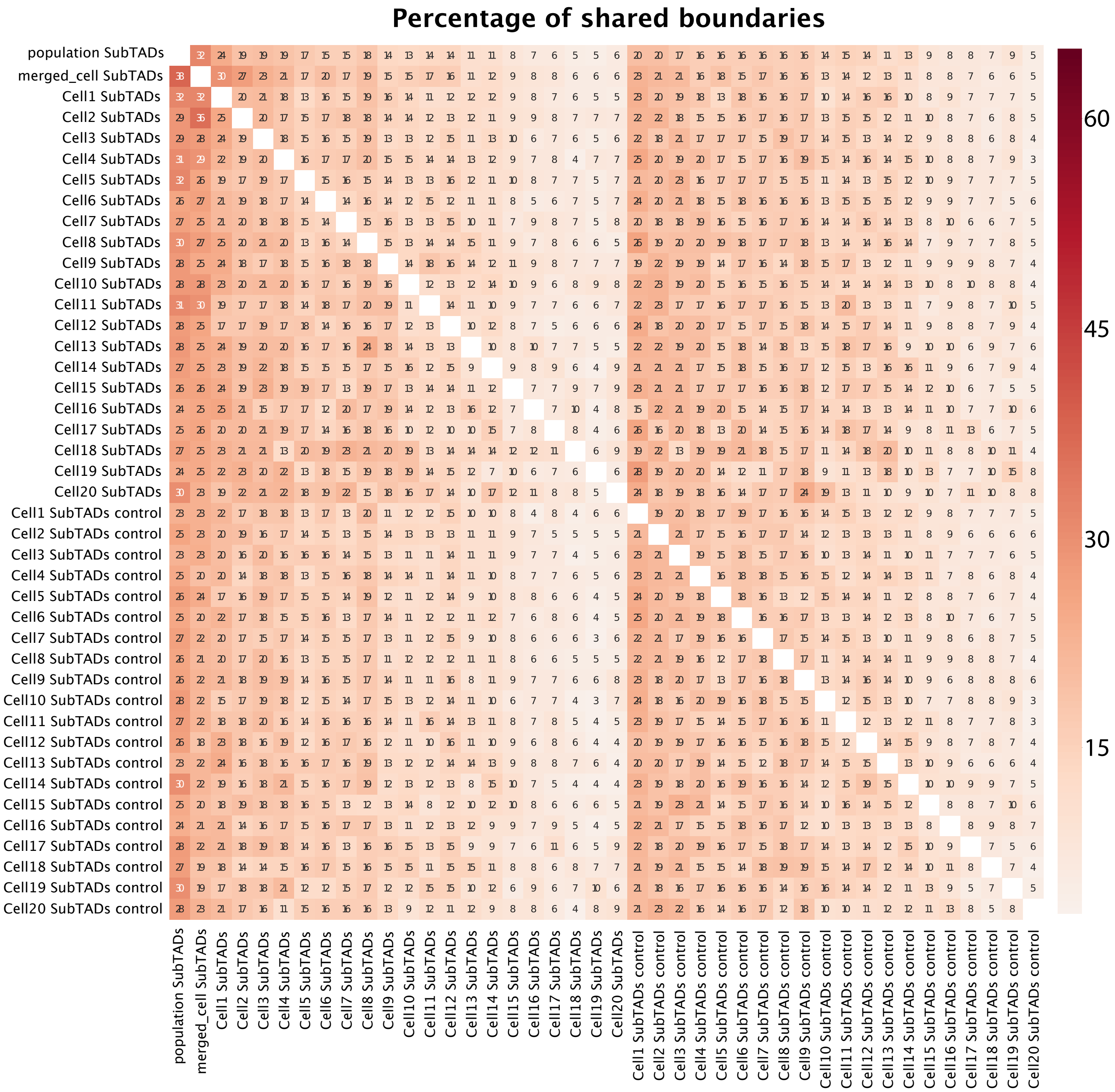

Supplementary Figure 14. Sub-TAD boundary profiles are not conserved between individual cells. Percentage of sub-TAD boundaries excluding TAD boundaries shared between *Drosophila* BG3 single cells, bulk BG3 *in situ* Hi-C, merged snHi-C data, and control shuffled datasets, pairwise comparisons. *P*-values of permutation tests for the sub-TAD boundaries were performed 1,000 times and are not shown. All of them are <0.01.

Supplementary Figure 15

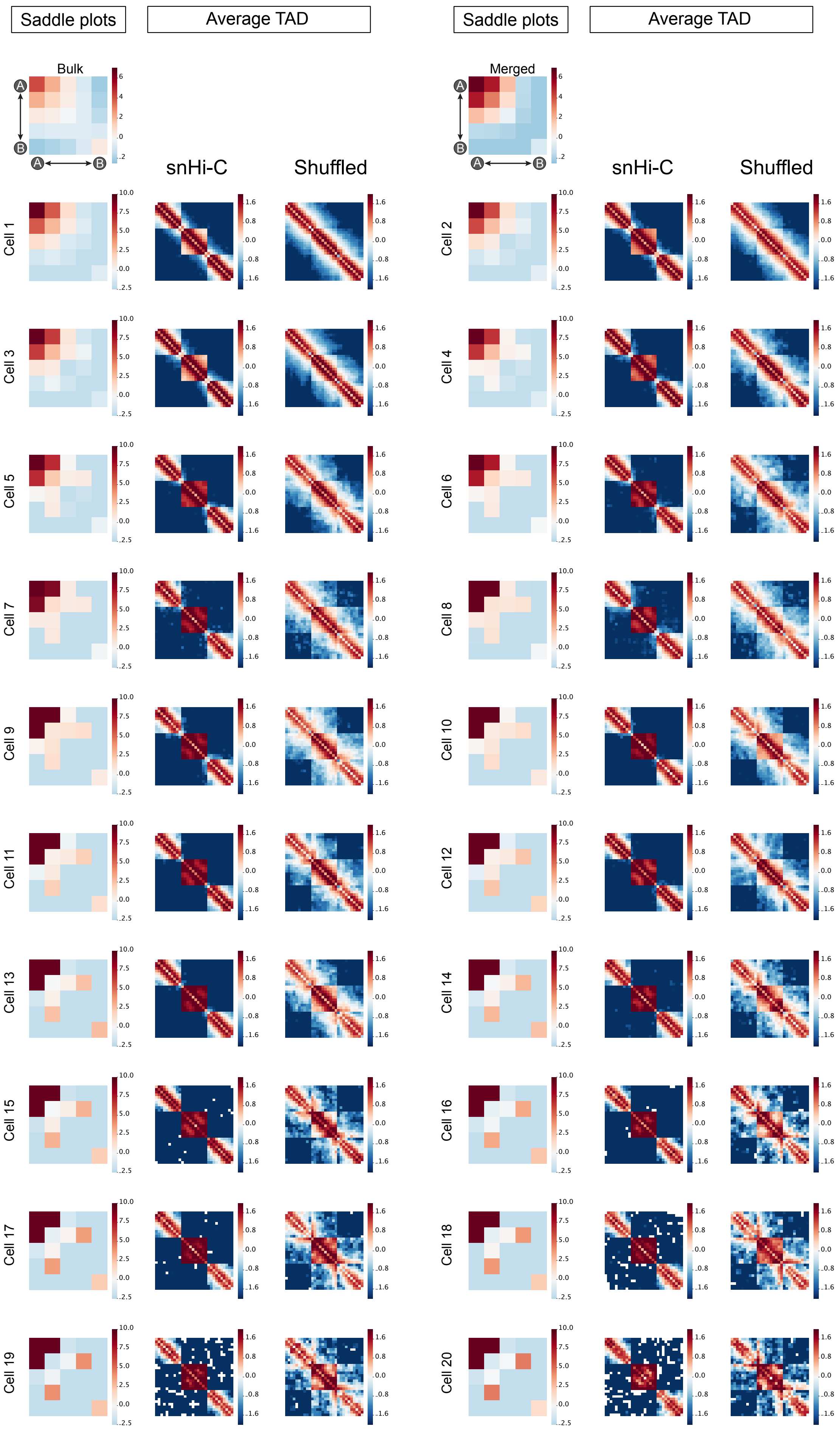

Supplementary Figure 15. Saddle plots and average TAD in individual cells.
Top: saddle plots for bulk Hi-C and merged contacts from all snHi-C datasets are shown on top. Below: saddle plot, average TAD plot, and average TAD plot for shuffled controls are displayed for each cell. Shuffled controls were obtained by the procedure described in Supplementary Fig. 4a-d. For saddle plots of bulk and merged data, log2 of observed over expected of iteratively corrected maps was used. For individual cells and saddle plot, log2 of observed over expected of pulled raw maps is shown. For individual cells and average TAD plots, log2 of pulled raw maps normalized by the mean number of contacts in a sliced window around TAD is provided.

Supplementary Figure 16

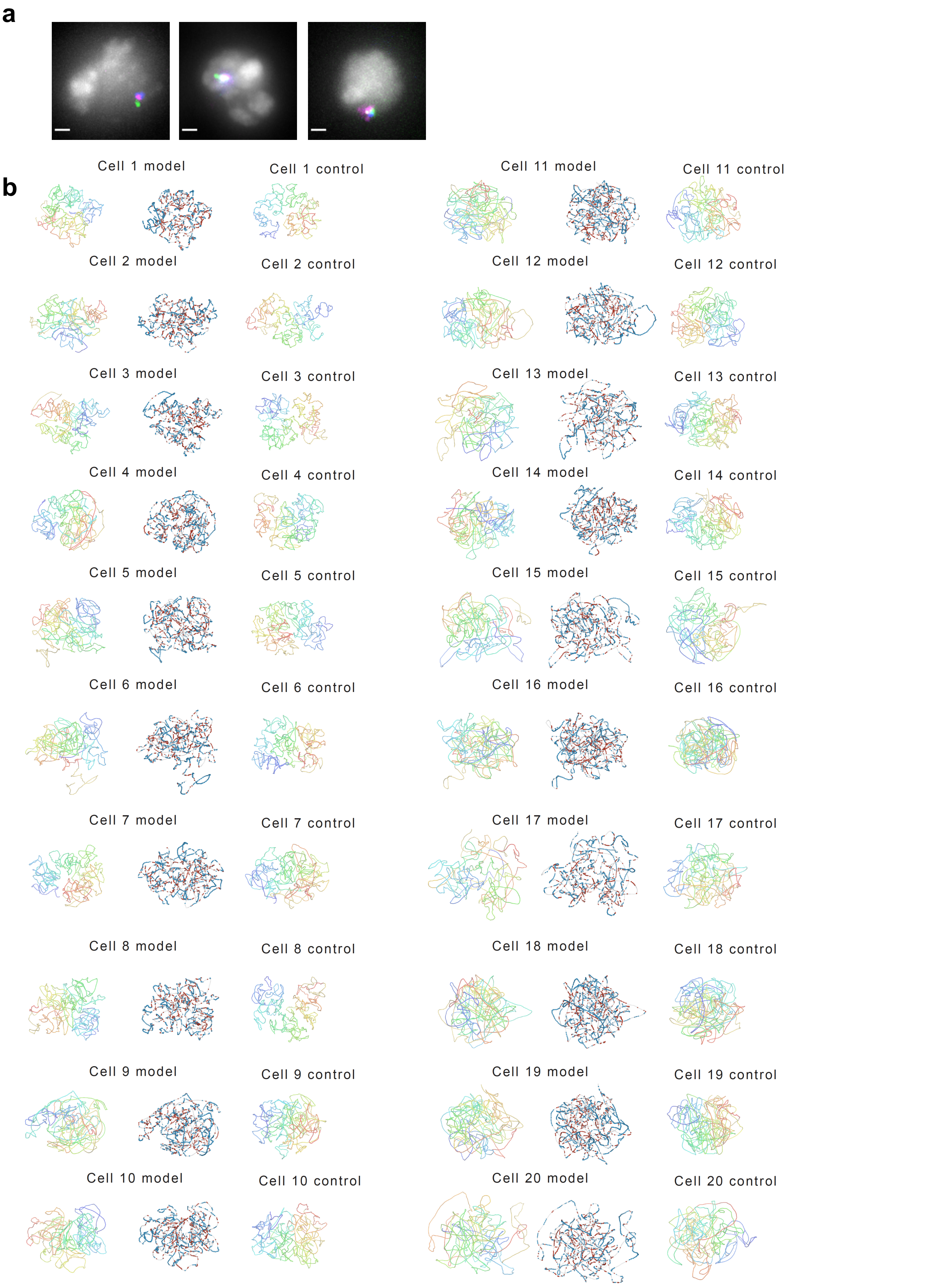

Supplementary Figure 16. Modeling of 3D folding of individual chromosomes.
(a) Examples of FISH images (related to Supplementary Fig. 19c) demonstrating that ChrX is actually haploid in the used batch of the BG3 cell line. Scale bar = 1 μm. **(b)** 3D models of individual X chromosomes obtained from real snHi-C contacts and from shuffled control snHi-C maps. The models are shown as a stick model; positions of each genomic bin were averaged in the sliding window of size 15 centered at each bin. The models obtained from real snHi-C contacts are colored by a rainbow approach and by chromatin colors (as described in Methods, red – active, dark grey – inactive). Source data are provided as a Source Data file.

Supplementary Figure 17

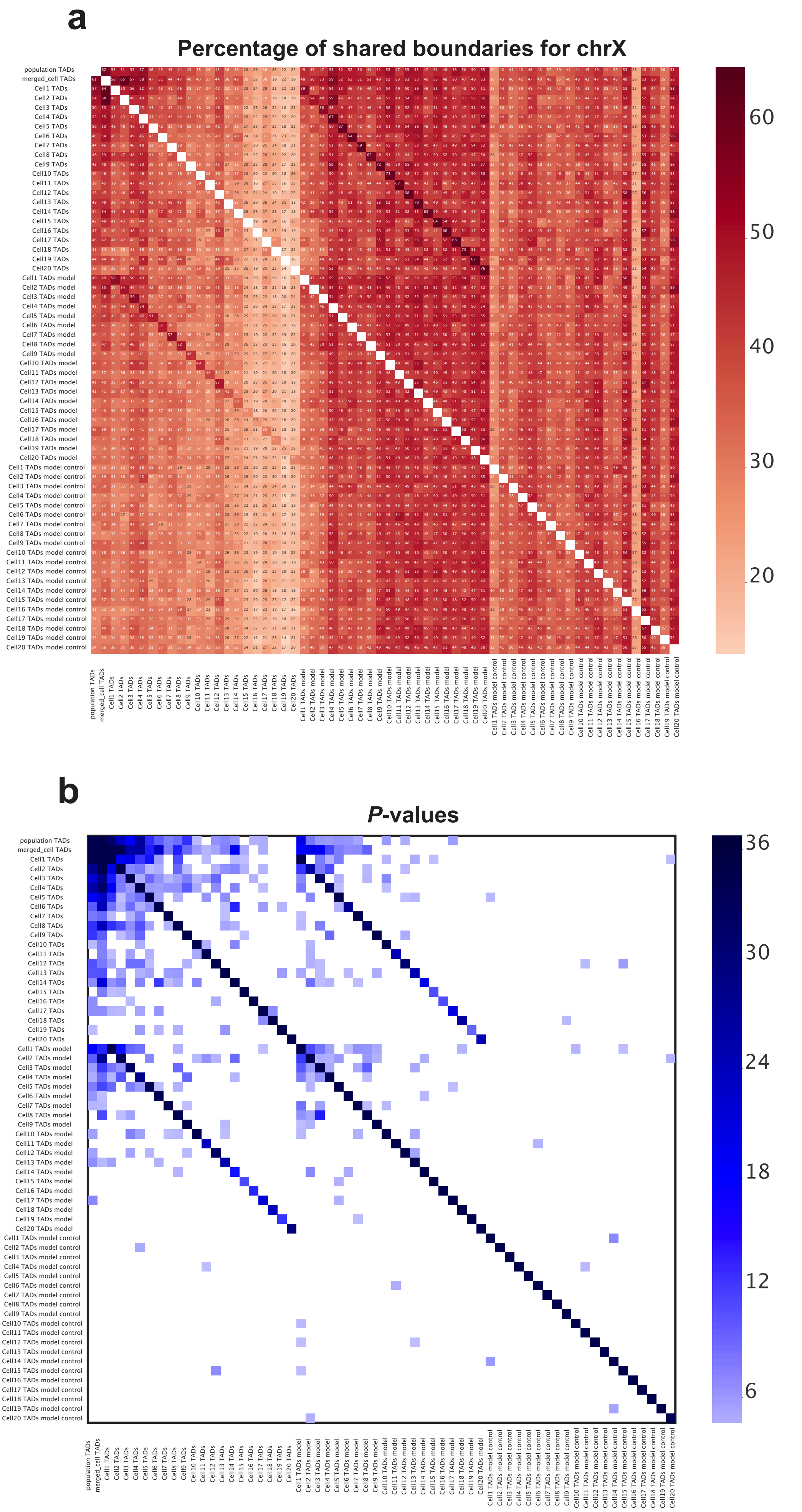

Supplementary Figure 17. TAD boundaries are reproduced in single-chromosome models.
(a) Percentage of TAD boundaries shared between individual cells, 3D models, and 3D models derived from shuffled snHi-C data (control). **(b)** *P*-values of permutation tests for TAD boundaries for (a).

Supplementary Figure 18

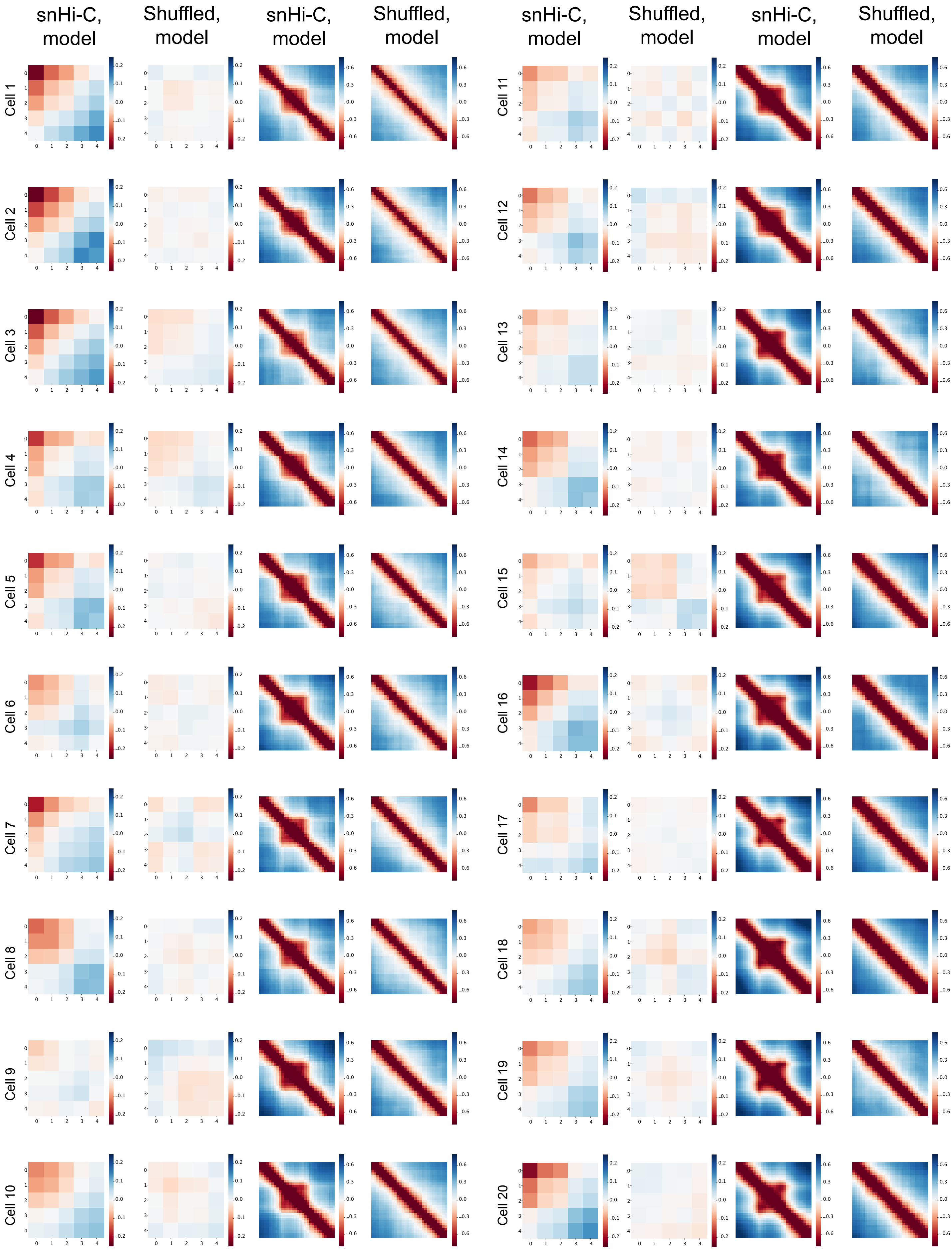

Supplementary Figure 18. Saddle plots and average TADs in individual models.
Four images in a row represent a single model of a chromosome X. For each model, there are saddle plots for real snHi-C modeling, saddle plots for shuffled controls, average TADs for snHi-C, and average TADs for control models. log2 values of observed over expected for average distance matrices were used; thus, smaller values represent a closer distance of corresponding bins of genome in 3D space.

Supplementary Figure 19

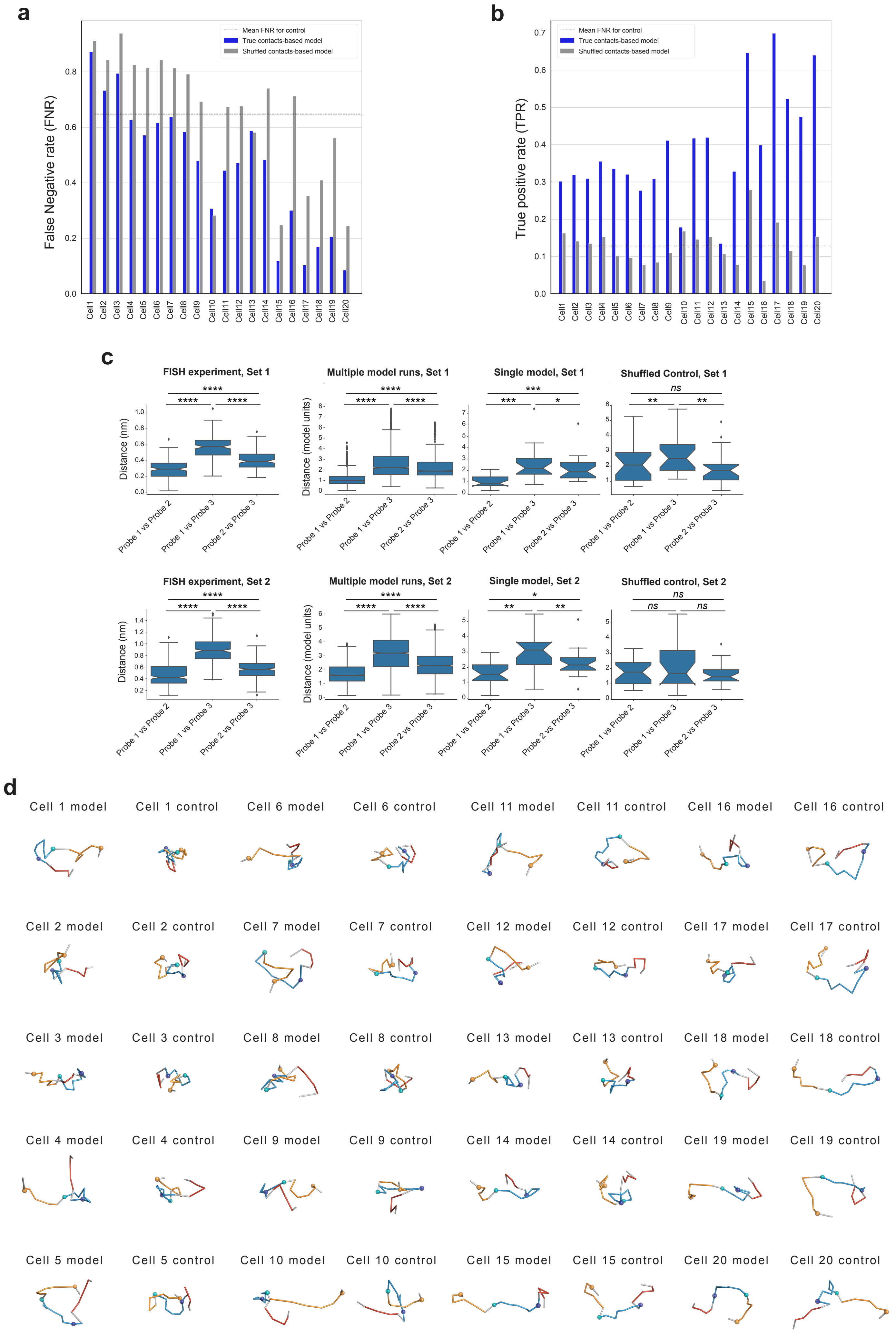

Supplementary Figure 19. Models recapitulate biologically meaningful contacts in single cells.
(a) False negative rate (FNR) of contacts obtained from models (real contacts and control are compared). Here, the set of true contacts for each cell is defined as the list of genomic bin pairs (10-kb resolution) with at least 1 interaction in the snHi-C map at a distance > 20 kb. The set of predicted contacts in the model is defined as the list of pairs of polymer monomers closer than 0.7 units based on average distance matrices. False negatives for the cell are defined as the number of pairs of bins that interact based on snHi-C but do not interact in the models by the criteria above. FNR is smaller for all the models based on real contacts except for Cells 10 and 13. Dotted line – mean FNR for the control; blue – true contacts-based model; grey – shuffled contacts-based model. **(b)** True positive rate (TPR) of contacts obtained from models. TPR is higher for all the models based on real contacts and is even higher than the mean TPR for the control shuffled contacts-based models. True positives for the cell are defined as the number of pairs of bins that interact based on snHi-C and also interact in the models by the criteria above. **(c)** Comparison of spatial distances between FISH probes in the DPD-simulated models (three right plots) and in situ FISH (left). Multiple model runs are 100 independent runs of DPD simulations. Single model and control are distance measurements for the models represented in Supplementary Fig. 16. Two independent sets of FISH probes were selected. ****$p < 0.0001$, ***$p < 0.001$, **$p < 0.01$, *$p < 0.05$, ns – non-significant difference in the two-sided Wilcoxon test. Set of probes 1: n = 132 independent measurements; Set of probes 2: n = 122 independent measurements.
Set 1:
Probe-1 = chrX:3,871,158..3,892,065 bp
Probe-2 = chrX:3,960,041..3,983,074 bp
Probe-3 = chrX:4,054,120..4,075,361;
Set 2:
Probe-1 = chrX:17,644,479..17,663,154 bp
Probe-2 = chrX:17,704,670..17,725,745 bp
Probe-3 = chrX:17,764,735..17,783,789.
Source data are provided as a Source Data file.
(d) Visualization of FISH probe regions (set 2) in models and conformation comparison with models obtained from real contacts and the shuffled control. Yellow, light-blue, and red stick fragments represent three TADs; three spheres represent the corresponding FISH probes.

Supplementary Figure 20

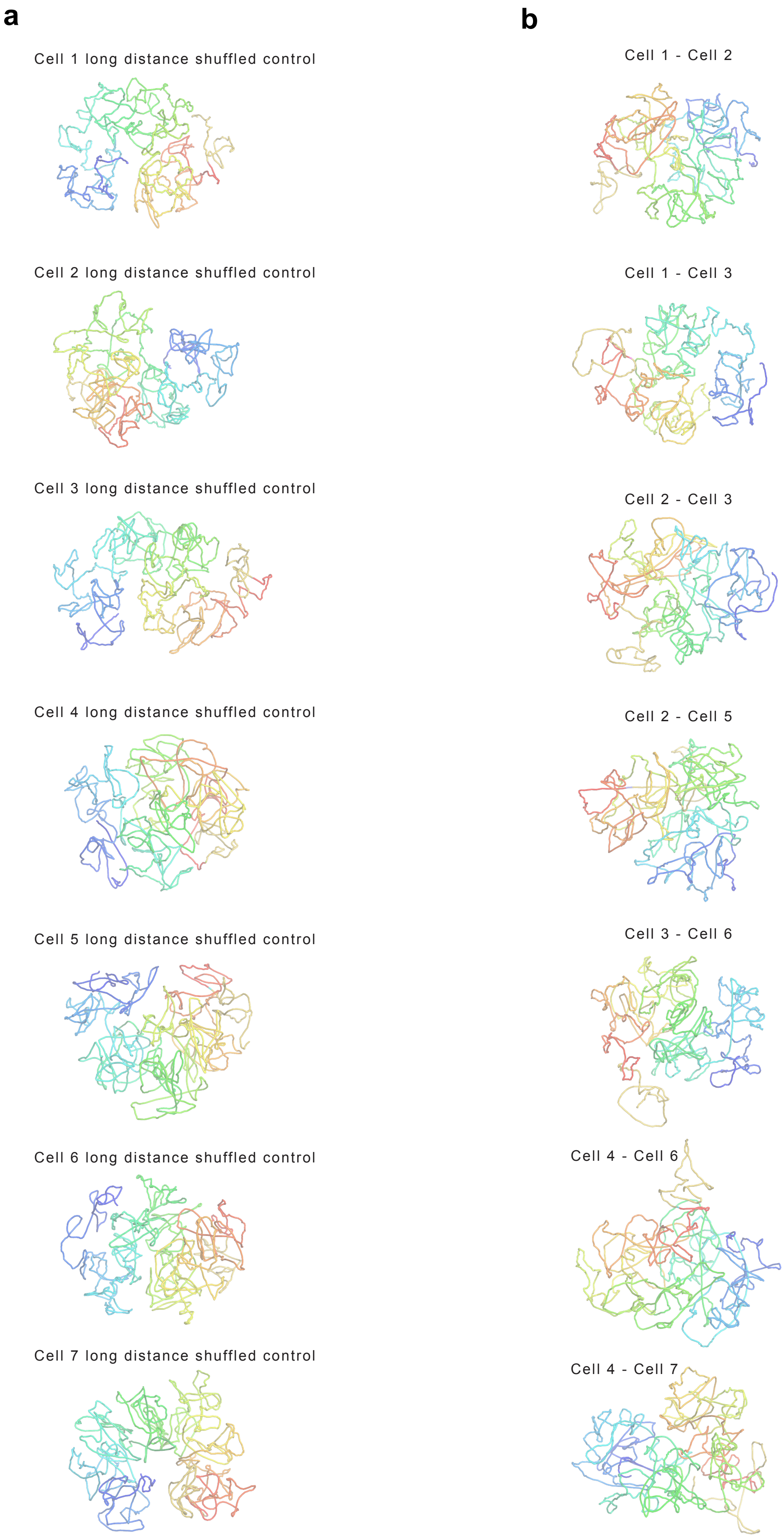

Supplementary Figure 20. 3D modeling controls.
(a) Models obtained for snHi-C interactions with shuffled contacts at a distance > 200 kb (long-distance shuffled control).
(b) Models obtained for snHi-C pairs of cells that were artificially merged and subsampled to the number of contacts of one of the cells. The number of contacts of the first cell in the pair was selected as a reference.

Supplementary Figure 21

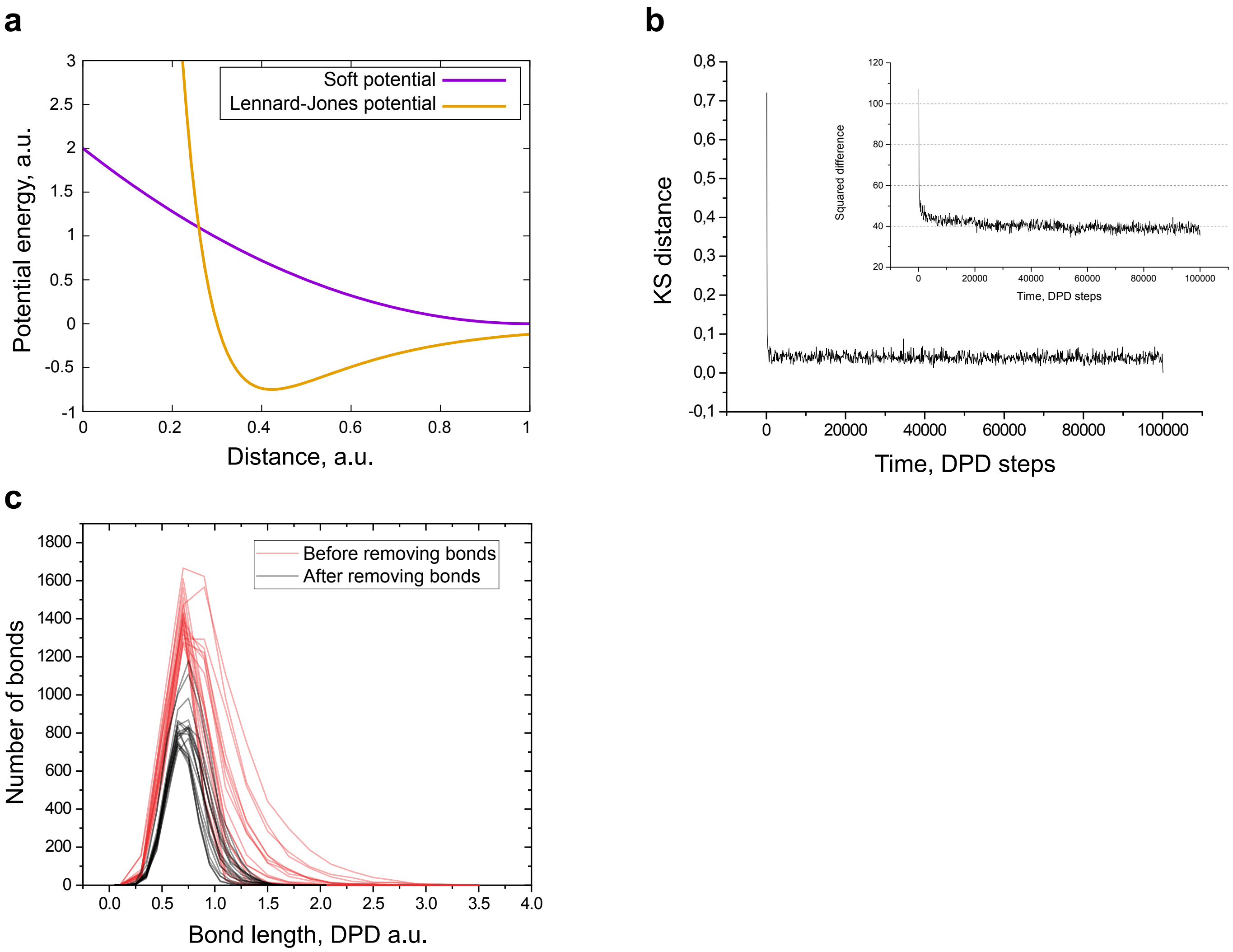

Supplementary Figure 21. DPD polymer simulations.
(a) Qualitative comparison of soft potential from DPD and Lennard-Jones potential (see Online Methods) from classical MD demonstrates a significant difference in behavior near zero. **(b)** Main graph is the dependency of Kolmogorov-Smirnov distance between distance matrices of final conformation and conformation at other timepoints. In the inset, there is the Euclidean norm of difference between two distance matrices: the final one and at other timepoints. Such tests revealed the fast relaxation of spatial conformation for the proposed reconstruction method. **(c)** Distribution of bonds' length before and after removing overstretched bonds. Despite the threshold for additional bonds being l<1.5, the length of the backbone bonds could be higher than 1.5. After removing additional bonds, heavy tails of the distributions "before" are not present.

Supplementary Table 1

Sequencing and mapping statistics for snHi-C datasets

Cell	Raw reads	Mapped reads	P (typical Hi-C) pairs	H (hops) pairs	J (junctions supported by restriction site) pairs	Removed duplicates	Number of unique contacts	Contacts from restriction fragments with > 4 contacts	Contacts from restriction fragments with > 4 contacts on chrX	Maximum number of contacts per restriction fragments	Maximum number of contacts per restriction fragments on chrX	Number of restriction fragments with > 4 contacts	Number of chr X restriction fragments with > 4 contacts	Final unique contacts	Final unique contacts on chrX
Cell1	44148251	26639477	23543936	507058	2588483	5322544	349051	1905	18	11	5	371	4	107823	11898
Cell2	68632913	39168691	33757160	1059441	4352090	7703393	160751	421	4	25	5	77	1	77770	8174
Cell3	34187865	19797650	17446338	374445	1976867	3071733	295915	368	11	8	6	70	4	73691	8408
Cell4	58947730	22197761	19741636	508544	1947581	6938793	193845	72	6	11	6	14	1	41439	4867
Cell5	50651907	31979756	26885818	892647	4201291	4047611	70297	314	7	13	11	63	5	38174	3917
Cell6	49705351	16513692	13628560	661470	2223662	3629266	94498	58	5	6	5	11	1	35420	3628
Cell7	43834863	13472471	10757036	489079	2226356	3858726	86946	194	13	10	8	39	5	30620	3348
Cell8	55049855	11567500	9733256	438663	1395581	2461501	145606	56	0	8	4	10	0	38019	3275
Cell9	78541387	65267034	55840373	2020682	7405979	7281553	50349	666	22	24	13	106	9	27059	2732
Cell10	41639146	8557235	7157042	376074	1024119	2137327	67414	41	15	16	5	6	3	24882	2585
Cell11	59065312	34086673	30192109	740740	3153824	10265078	129935	229	1	20	5	41	1	25483	2558
Cell12	97406463	41318464	33589103	5018090	2711271	6957870	132613	155	18	37	10	19	3	27215	2988
Cell13	52867991	38130313	33034704	1502780	3592829	8718835	118149	201	9	10	10	37	5	22714	2344
Cell14	68676167	18782199	12614794	2894410	3272995	4745700	78900	163	2	11	11	31	3	21541	2046
Cell15	15004439	755908	661306	18173	76429	141119	58575	0	0	4	3	0	0	14919	1448
Cell16	58244415	22185479	16514468	872321	4798690	7708534	69613	388	7	29	29	60	7	14325	1168
Cell17	22231710	17614914	15932015	320181	1362718	2931803	47619	16	0	6	3	3	0	11087	1282
Cell18	38703945	22557412	10679605	597650	11280157	15133641	42358	453	10	29	7	76	12	12534	929
Cell19	59894379	34471518	28505516	602827	5363175	10805432	58616	280	1	12	10	59	3	11688	1002
Cell20	24336293	6568388	3720850	61977	2785561	4022939	24282	37	0	8	2	7	0	8032	765

Supplementary Table 2

Number of removed bonds. Green means number of the removed contacts in the shuffled models greater than in experimental data, red is the opposite.				
Cell ID	Experimental data	Shuffled data	Difference between shuffled and experimental data	Shuffled on the long genomic distances only
1	1075	64	-1011	1271
2	578	712	134	654
3	640	1285	645	1257
4	171	268	97	236
5	224	438	214	
6	190	486	296	482
7	208	503	295	451
8	230	552	322	471
9	59	128	69	
10	20	61	41	
11	38	214	176	
12	0	0	0	
13	13	248	235	
14	85	175	90	
15	8	166	158	
16	0	0	0	
17	0	2	2	
18	5	82	77	
19	2	25	23	
20	0	0	0	

Supplementary Table 3

Number of removed contacts if contacts sampled from two cells. Green means that number of the removed contacts in the model higher than in case of each cell of the mixture.	
Cell IDs	Number of removed bonds
Cell4 - Cell8	353
Cell4 - Cell7	299
Cell1 - Cell2	1146
Cell1 - Cell3	1238
Cell2-Cell7	703
Cell2-Cell3	703
Cell3-Cell6	893
