# Supplementary material for: Order and stochasticity in the folding of individual Drosophila genomes
Source: Nat Commun. 2021 Jan 4;12:41. doi: 10.1038/s41467-020-20292-z (PMC7782554; doi:10.1038/s41467-020-20292-z)
Supplement: Supplementary file 3 — Reporting Summary [file 41467_2020_20292_MOESM3_ESM.pdf]

nature research

Corresponding author(s): Sergey V. Razin

Last updated by author(s): Sep 21, 2020

Reporting Summary

Nature Research wishes to improve the reproducibility of the work that we publish. This form provides structure for consistency and transparency in reporting. For further information on Nature Research policies, see our Editorial Policies and the Editorial Policy Checklist.

Statistics

For all statistical analyses, confirm that the following items are present in the figure legend, table legend, main text, or Methods section.

n/a	Confirmed	
☐	☒	The exact sample size (n) for each experimental group/condition, given as a discrete number and unit of measurement
☐	☒	A statement on whether measurements were taken from distinct samples or whether the same sample was measured repeatedly
☐	☒	The statistical test(s) used AND whether they are one- or two-sided<br>*Only common tests should be described solely by name; describe more complex techniques in the Methods section.*
☒	☐	A description of all covariates tested
☒	☐	A description of any assumptions or corrections, such as tests of normality and adjustment for multiple comparisons
☐	☒	A full description of the statistical parameters including central tendency (e.g. means) or other basic estimates (e.g. regression coefficient) AND variation (e.g. standard deviation) or associated estimates of uncertainty (e.g. confidence intervals)
☐	☒	For null hypothesis testing, the test statistic (e.g. F, t, r) with confidence intervals, effect sizes, degrees of freedom and P value noted<br>*Give P values as exact values whenever suitable.*
☒	☐	For Bayesian analysis, information on the choice of priors and Markov chain Monte Carlo settings
☒	☐	For hierarchical and complex designs, identification of the appropriate level for tests and full reporting of outcomes
☒	☐	Estimates of effect sizes (e.g. Cohen's d, Pearson's r), indicating how they were calculated

Our web collection on statistics for biologists contains articles on many of the points above.

Software and code

Policy information about availability of computer code

Data collection

FISH image capture was performed using Micromanager 1.4 (https://open-imaging.com/). Hardware control and image capture were carried out using µManager88. Images were deconvolved using Nikon NIS-Elements. Measurements were taken using Imaris 8.2.

Data analysis

The data processing pipeline is available at https://github.com/agalitsyna/sc_dros. The modeling pipeline is available at https://github.com/polly-code/DPD_withRemovingBonds.

We developed a custom approach for snHi-C data processing termed ORBITA (One Read-Based Interaction Annotation), as described below.

1. Reads mapping.
As the first step of the approach, FASTQ files with paired-end sequencing data are mapped to Drosophila reference genome dm3 using Burrows-Wheeler Aligner (BWA-MEM, console version 0.7.17-r1188) with default parameters. Notably, this mapping procedure allows independent alignment of chimeric parts of both forward and reverse reads. This step results in BAM files with paired-end mapping information.

2. Annotated pairs retrieval.
In the next step, the BAM files are parsed with an adapted version of pairtools (https://github.com/mirnylab/pairtools) with our newly implemented option ORBITA. Among many other utilities for Hi-C data processing, we selected pairtools from the Mirny lab as the basis of our approach, due to the convenience and modular structure of its code. This version of the tool can be accessed at the GitHub repository https://github.com/agalitsyna/pairtools.
ORBITA treats each read in the BAM file independently, regardless of whether it is forward or reverse. Reads that are uniquely mapped to a single location of the genome are marked as type P, meaning that they are part of a standard Hi-C Pair with no DNA breakpoint evidence. Reads that contain precisely two successive regions uniquely mapped to different genomic locations (MAPQ>1) are selected for further DNA

April 2020

breakpoint annotation. ORBITA takes the genome restriction annotation (provided as a BED file with DpnII restriction fragments positions, produced by cooler diges) and compares each breakpoint against the list of restriction sites. For each 3'-end of the right chimeric part and 5'-end of the left chimeric part (in other words, ligated ends), both upstream and downstream restriction sites are annotated, and the distance to the closest one is calculated. If both ends are located sufficiently close (<10 bp) to any restriction site in the genome, ORBITA considers them as a true ligation junction of restricted fragments in the snHi-C proximity ligation step. These cases are marked as J type (ligation Junction), with the evidence of traversing the ligation junction of DpnII restriction fragments. If at least one ligated end of the chimeric read was not mapped to the restriction site, ORBITA marks it as H (template switch, or Hopping of Phi29 DNA polymerase). To simplify the ORBITA approach, we omit the cases with more complicated scenarios of read mapping, when three or more uniquely mapped chimeric parts of a single-end read were present. If the read contains multiple mapped chimeric parts, it is discarded. ORBITA produces the resulting PAIRS file with annotation of JJ pairs (with the evidence of the ligation) that are accepted for further processing. If not explicitly mentioned, the generic names "pair" or "contact" are used for snHi-C contacts with the evidence of the ligation junction.

3. Amplification duplicates removal
In the next step, we performed a correction for amplified duplicates of snHi-C contacts. Standard Hi-C uses amplification by the Illumina PCR protocol with primers that are ligated to the ends of sheared DNA. Thus, two independent Hi-C pairs can be PCR duplicates if their mapping positions coincide (e.g., see hiclib). However, the amplification in snHi-C is followed by sonication, resulting in random breaks of ligated DNA fragments. Hence, coinciding mapping positions cannot be used as a criterion of PCR duplication. Notably, we cannot distinguish the amplified pair contacting restriction fragments from the contacts of the same regions in the homologous chromosomes. Thus, we removed all multiple copies of restriction fragment pairs and retained unique contacts for each combinatorial pair of restriction fragments.

4. Fragment filtration
In the next step, we used restriction fragment filtration to reduce the possible contribution of copy number variation, read misalignment, and Phi29 DNA polymerase template switch that had not been removed by the ORBITA filter.
In theory, each restriction fragment of DNA has two ends and is present twice in the diploid nucleus of ML-DmBG3-c2 Drosophila cells; thus, we expect the upper limit of four unique contacts per restriction fragment if no unannotated genomic rearrangements, mismappings, or template switches occurred. For each restriction fragment, we calculated the observed number of contacts and removed fragments that had more than four contacts.
Before contact filtration by this rule, we compared the number of restriction fragments with more than four unique contacts according to ORBITA and one previous approach, hiclib for Flyamer et al. 2017. We obtained datasets for mouse nuclei from Flyamer et al. 2017 and Nagano et al. 2017 and mapped with the hiclib and ORBITA pipelines. We found a significant reduction in the number of unique contacts per fragment for snHi-C from Phi29 DNA polymerase datasets (Flyamer et al. 2017, present work], but not for scHi-C without Phi29 DNA polymerase (Nagano et al. 2017) (Supplementary Fig. 2, 3). Thus, we conclude that ORBITA is an effective approach to reduce the number of snHi-C artefactual contacts arising from random template switches of Phi29 DNA polymerase.

snHi-C interaction map construction
The resulting pair data were binned at 1 kb, 10 kb, 20-kb, 40-kb and 100-kb resolutions with cooler version 0.8.5 and stored in the COOL format. The HiGlass server was used for data visualization.

For bulk BG3 in situ Hi-C (two biological replicates), reads were mapped to Drosophila reference genome dm3 with Burrows-Wheeler Aligner (BWA-MEM, console version 0.7.17-r1188) with default parameters. For consistency with the snHi-C analysis, the resulting BAM files were parsed with pairtools v0.3.0, (https://github.com/mirnylab/pairtools) using default parameters. The resulting files were sorted by the pairtools module "sort"; replicates were merged by the pairtools module "merge" and duplicates were removed, allowing one mismatch between possible duplicates (pairtools dedup with --max-mismatch 1 and —mark-dups options). The resulting PAIRS file was binned with cooler at the same resolutions as the single-cell datasets. To remove the contribution of possible Hi-C technical artifacts, such as backward ligation, dangling ends, self-circles, and mirror reads, the first two diagonals of Hi-C maps were removed. As the last step of bulk Hi-C processing, the maps were iteratively corrected for the removal of coverage bias by the cooler balance tool with default parameters.
For the reproducibility control, both replicates were converted to interaction maps independently by the above pipeline. The resulting maps demonstrated a correlation of 0.9–0.95 as estimated by the HiCRep stratum-adjusted correlation coefficient for intrachromosomal maps smoothed with one-bin offset and genomic distance up to 300 kb at 20 kb resolution.

TAD calling in snHi-C and bulk BG3 in situ Hi-C data
We used Hi-C map segmentation with lavaburst (v0.2.0) (https://github.com/nvictus/lavaburst) with the modularity scoring function for TAD calling in Hi-C maps at 10-kb resolution.

Compartment annotation in snHi-C and bulk BG3 in situ Hi-C
For compartment annotation in bulk BG3 in situ Hi-C, we used eigenvector decomposition of cis-interactions maps for each chromosome, as implemented in cooltools call-compartments tool version 0.2.0 (https://github.com/mirnylab/cooltools).

Epigenetic analysis of TAD boundaries
We plotted the ChIP-chip signal around different types of boundaries with pybbi utility (https://github.com/nvictus/pybbi.git) based on UCSC tools.

Visualization of epigenetic states
The visualization was performed using the pymol software v. 2.3.2 (https://pymol.org/2/). 1D epigenetic data were added to the structure as a bead type and represented with a corresponding color. Analysis of different epigenetic states was performed via Python scripts (https://github.com/polly-code/DPD_withRemovingBonds).

For manuscripts utilizing custom algorithms or software that are central to the research but not yet described in published literature, software must be made available to editors and reviewers. We strongly encourage code deposition in a community repository (e.g. GitHub). See the Nature Research guidelines for submitting code & software for further information.

Data

Policy information about availability of data

All manuscripts must include a data availability statement. This statement should provide the following information, where applicable:

- Accession codes, unique identifiers, or web links for publicly available datasets
- A list of figures that have associated raw data
- A description of any restrictions on data availability

Raw and processed snHi-C and bulk BG3 in situ Hi-C data are available in the GEO NCBI under accession number GSE131811.

List of publicly available GEO sources used in this study: GSE122603 (Hi-C for Kc167 and BG3 cell lines for comparison of stable TAD boundaries), GSE58821 (MSL; ChIP-seq), GSE69013 (RNA-Seq).
List of publicly available modENCODE data sources used in this study: total RNA of ML-DmBG3-c2 cell line assessed by RNA tiling array (modENCODE id 713) and the ChIP-chip for MOF (id 3041), BEAF-32 (id 921), Chriz (275), CP190 (924), CTCF (3280), dmTopo-II (5058), GAF (2651), H1 (3299), HP1a (2666), HP1b (3016), HP1c (942), HP2 (3026), HP4 (4185), ISWI (3030), JIL-1 (3035), mod(mdg4) (324), MRG15 (3045), NURF301 (5063), Pc (325), RNA-polymerase-II (950), Su(Hw) (951), Su (var)3-7 (2671), Su(var)3-9 (952), WDS (5148), H3 (3302), H3K27ac (295), H3K27me3 (297), H3K36me1 (299), H3K36me3 (301), H3K4me1 (2653), H3K4me3 (967), H3K9me2 (310), H3K9me3 (312), H4K16ac (316). dRING binding data were obtained from modENCODE as a ChIP-chip normalized array file (ID 927 54).

Field-specific reporting

Please select the one below that is the best fit for your research. If you are not sure, read the appropriate sections before making your selection.

- [x] Life sciences
- [ ] Behavioural & social sciences
- [ ] Ecological, evolutionary & environmental sciences

For a reference copy of the document with all sections, see nature.com/documents/nr-reporting-summary-flat.pdf

Life sciences study design

All studies must disclose on these points even when the disclosure is negative.

Sample size

snHi-C libraries were prepared in three independent experiments to ensure the robustness of the snHi-C procedure and reproducibility of the data quality.

Data exclusions

We obtained filtered contacts for 88 individual nuclei after the initial round of sequencing. Before the second round of sequencing, we assessed the robustness of the number of unique contacts by subsampling of raw datasets (Supplementary Fig. 2a). For each library, we created a uniform grid of sequencing depth (from 0 to the resulting number of reads with the step of 100,000 reads). We then randomly selected X reads from the full library and calculated the number of unique contacts (as described above) for each number from the grid X. We repeated this procedure ten times and plotted the mean number of unique contacts for each sequencing depth from the grid.
We proposed that there are a significant number of cells containing PCR duplicates and that the number of contacts increases slowly depending on the sequencing depth due to the poor efficiency of the snHi-C protocol. Further sequencing of these cells would result in a relatively small improvement of the detectable number of unique contacts. The number of contacts for other cells increases more rapidly with the number of reads but reaches a plateau once the maximum number of unique contacts is achieved. Thus, additional sequencing of these cells might result in reading duplicated contacts.
For other cells, the number of contacts grew slowly with sequencing depth (Supplementary Fig. 2a). However, for all these cells, the number of unique contacts gradually increased with no plateau signature. We selected the cells displaying the best growth of the number of contacts, indicative of the good quality of the dataset. The top 20 cells by the number of unique contacts were subjected to an additional round of sequencing. The same mapping and parsing pipeline was used for these datasets. Technical replicates (initial and additional rounds of snHi-C libraries sequencing) were merged at the annotated PAIRS file stage.

Replication

All three batches of snHi-C experiments were performed independently

Randomization

The samples were not randomly allocated into experimental groups because every sample consisted of cells of the same cell type taken into experiment from the same biological and growth conditions.

Blinding

The investigators were not blinded during data collection and analysis because the snHi-C experiments were performed on the untreated cells grown in the same biological and culture conditions.

Reporting for specific materials, systems and methods

We require information from authors about some types of materials, experimental systems and methods used in many studies. Here, indicate whether each material, system or method listed is relevant to your study. If you are not sure if a list item applies to your research, read the appropriate section before selecting a response.

Materials & experimental systems

n/a	Involved in the study
☒	☐ Antibodies
☐	☒ Eukaryotic cell lines
☒	☐ Palaeontology and archaeology
☒	☐ Animals and other organisms
☒	☐ Human research participants
☒	☐ Clinical data
☒	☐ Dual use research of concern

Methods

n/a	Involved in the study
☒	☐ ChIP-seq
☐	☒ Flow cytometry
☒	☐ MRI-based neuroimaging

Eukaryotic cell lines

Policy information about cell lines

Cell line source(s)	ML-DmBG3-c2 from the collection of Institute of molecular genetics RAS
Authentication	The cell line was not authentificated
Mycoplasma contamination	Cell culture is mycoplasma-free (PCR-tested)
Commonly misidentified lines (See ICLAC register)	No commonly misidentified cell lines were used in this study.

Flow Cytometry

Plots

Confirm that:

☒ The axis labels state the marker and fluorochrome used (e.g. CD4-FITC).

☒ The axis scales are clearly visible. Include numbers along axes only for bottom left plot of group (a 'group' is an analysis of identical markers).

☒ All plots are contour plots with outliers or pseudocolor plots.

☒ A numerical value for number of cells or percentage (with statistics) is provided.

Methodology

Sample preparation	We modified the previously published single-nucleus Hi-C protocol as follows: 5–10 million cells were fixed in 1× phosphate-buffered solution (PBS) with 2% formaldehyde for 10 min with occasional mixing. The reaction was stopped by the addition of 2 M glycine to give a final concentration of 125 mM. Cells were centrifuged (1,000 x g, 10 min., 4 °C), resuspended in 50 µl of 1× PBS, snap-frozen in liquid nitrogen, and stored at -80 °C. Defrozen cells were lysed in 1.5 ml isotonic buffer (50 mM Tris-HCl pH 8.0, 150 mM NaCl, 0.5% (v/v) NP-40 substitute (Fluka), 1% (v/v) Triton-X100 (Sigma), 1× Halt™ Protease Inhibitor Cocktail (Thermo Scientific) on ice for 15 min. Cells were centrifuged at 2,500 x g for 5 min, resuspended in 100 µl of 1× DpnII buffer (NEB), and pelleted again. The pellet was resuspended in 200 µl of 0.3% SDS in 1.1× DpnII buffer and incubated at 37 °C for 1 hour. Then, 330 µl of 1.1× DpnII buffer and 53 µl of 20% Triton X-100 (Sigma) were added, and the suspension was incubated at 37 °C for 1 hour. Next, 600 U of DpnII enzyme (NEB) were added, and the chromatin was digested overnight (14–16 hours) at 37 °C with shaking (1,400 rpm). On the following day, 200 U of DpnII enzyme were added, and the cells were incubated for an additional 2 hours. DpnII was then inactivated by incubation at 65 °C for 20 min. Nuclei were centrifuged at 3,000 x g for 5 min, resuspended in 100 µl of 1× T4 DNA ligase buffer (Fermentas), and pelleted again. The pellet was resuspended in 400 µl of 1× T4 DNA ligase buffer, and 75 U of T4 DNA ligase (Fermentas) were added. Chromatin fragments were ligated at 16 °C for 6 hours. Next, the nuclei were centrifuged at 5,000 x g for 5 min, resuspended in 100 µl of sterile 1× PBS, stained with Hoechst, and single nuclei were isolated into wells of a standard 96-well PCR plate (Thermo Fisher) using FACS (BD FACSAriaTMIII). Each well contained 3 µl of sample buffer from the Illustra GenomiPhi v2 DNA amplification kit (GE Healthcare).
Instrument	BD FACSAriaTMIII
Software	DIVA (Beckton-Dickinson)
Cell population abundance	The aim of the experiment was to sort and isolate single fixed cell nuclei into 96-well plates. To verify the purity and the presence of one nucleus per well, the content of two wells was inspected under the fluorescence microscope. One nucleus per well and the absence of cell/nuclear debris was seen in both cases.
Gating strategy	Forward scatter area (FSC-A) vs. side scatter area (SSC-A) plot was used to gate the DAPI-stained single nuclei population and remove the debris.

☒ Tick this box to confirm that a figure exemplifying the gating strategy is provided in the Supplementary Information.
